# Human Recombinant DNase I (Pulmozyme^®^) Inhibits Lung Metastases in Murine Metastatic B16 Melanoma Model That Correlates with Restoration of the DNase Activity and the Decrease SINE/LINE and c-Myc Fragments in Blood Cell-Free DNA

**DOI:** 10.3390/ijms222112074

**Published:** 2021-11-08

**Authors:** Ludmila Alekseeva, Aleksandra Sen’kova, Innokenty Savin, Marina Zenkova, Nadezhda Mironova

**Affiliations:** Institute of Chemical Biology and Fundamental Medicine SB RAS, Lavrentiev ave., 8, 630090 Novosibirsk, Russia; mila_alex@ngs.ru (L.A.); alsenko@mail.ru (A.S.); kesha_savin@mail.ru (I.S.); marzen@niboch.nsc.ru (M.Z.)

**Keywords:** DNase I, Pulmozyme^®^, drug repurposing, circulating cell-free DNA, neutrophil extracellular traps, SINE elements, LINE elements, tumor, metastasis

## Abstract

Tumor-associated cell-free DNAs (cfDNA) play an important role in the promotion of metastases. Previous studies proved the high antimetastatic potential of bovine pancreatic DNase I and identified short interspersed nuclear elements (SINEs) and long interspersed nuclear elements (LINEs)and fragments of oncogenes in cfDNA as the main molecular targets of enzyme in the bloodstream. Here, recombinant human DNase I (commercial name Pulmozyme^®^), which is used for the treatment of cystic fibrosis in humans, was repurposed for the inhibition of lung metastases in the B16 melanoma model in mice. We found that Pulmozyme^®^ strongly reduced migration and induced apoptosis of B16 cells *in vitro* and effectively inhibited metastases in lungs and liver *in vivo*. Pulmozyme^®^ was shown to be two times more effective when administered intranasally (i.n.) than bovine DNase I, but intramuscular (i.m.) administration forced it to exhibit as high an antimetastatic activity as bovine DNase I. Both DNases administered to mice either i.m. or i.n. enhanced the DNase activity of blood serum to the level of healthy animals, significantly decreased cfDNA concentrations, efficiently degraded SINE and LINE repeats and c-Myc fragments in the bloodstream and induced apoptosis and disintegration of neutrophil extracellular traps in metastatic foci; as a result, this manifested as the inhibition of metastases spread. Thus, Pulmozyme^®^, which is already an approved drug, can be recommended for use in the treatment of lung metastases.

## 1. Introduction

Drug repurposing refers to a general approach in which a drug approved for the treatment of certain pathological states is tested against other diseases [1]. This approach is critical as it takes advantage of drugs with well-known pharmacokinetics, pharmacodynamics, toxicity profiles and even predictable dosages, as well as other investigated factors, such as side effects and the possibility of combination with other drugs. Thus, the chance of their failure in the future decreases, and the cost of their development and the time required for their approval are reduced [2]. The commercial aspect is also important, since drug repurposing provides quick access to drugs with already established production lines and supply chains, thus facilitating the discovery process [3]. As a result, the cost and time required for preclinical screening can be significantly reduced. The growing attention to this strategy is strongly dependent on the ability to obtain accurate mechanistic views of the repurposed drug. Indeed, it is on the basis of this information that the selected medication can be changed for a new indication. Currently, drug repurposing is urgently being performed to find new approaches to treat rapidly spreading COVID-19 [3] andmost widely used in the search for new anticancer and antimetastatic drugs [4].

Dornase alfa, a recombinant form of human deoxyribonuclease I (DNase I), is a class B drug that has been successfully used for the treatment of cystic fibrosis [5]. It has a very low toxicity due to its short half-life and poor systemic absorption [6]. Today, dornase alfa in the form of the commercially available medication Pulmozyme^®^ is recommended for the therapy of cystic fibrosis, especially for children, as well as for pleural effusion and emphysema [7]. Dornase alfa was found to degrade (fDNA)integrated in neutrophil extracellular traps (NETs), which are formed under various infectious and autoimmune diseases [8]. The first clinical trials tested the effects of nebulised dornase in mechanically ventilated patients with severe COVID-19 or severe trauma in an attempt to reduce NET-related respiratory failure [9]. Also, attempts have been made to use dornase alfa to relieve acute symptoms in patients with severe lung cancer [10]. However, so far there have been no reliable studies on the possible role of dornase alfa as an anticancer drug.

The antimetastatic potential of bovine pancreatic DNase I was shown for the first time on a model of spontaneous lymphocytic leukemia in AKP mice and on the L5178Y ML mouse tumor model [11,12]. Now, the antimetastatic activity of DNase I has been proven in a number of mouse tumor models; however, there are only a few studies where correlations were found between the decrease in the number of metastases, the decrease in blood cfDNA levels and the increase in DNase activity in the blood plasma [13,14,15,16,17,18,19,20]. Molecular targets of DNase I in the bloodstream of mice with tumors were revealed to be fragments of oncogenes (c-Myc, Kras, Hmga) and tandem repeats, including mobile genetic elements (MGE) [19,20]. It is assumed that metastasis can occur through the transfection of normal cells by circulating cfDNA. An increase in the total number of the oncogene fragments *MYCN*, *HER2* and *EGFR*, as well as MGE, in the cfDNA in the blood of patients with oncological diseases was noted in a number of studies [21,22,23]. MGE often found in the cfDNA pool are SINE- and LINE-elements (in particular, Alu and LINE1) [24,25,26], and it is assumed that MGE can play a key role in malignant transformation of cells, since some MGE retain the ability to move along the genome [27]. According to the type of transposition, MGE are divided into two classes: DNA transposons, which use the “cut and paste” method, and retrotransposons, mediated by RNA reverse transcription. Unlike SINEs, LINEs, encoding reverse transcriptase and endonuclease are independent mobile elements. SINEs can be transposed only with LINEs.

Some data have been accumulated on the role of SINE and LINE elements in carcinogenesis [28,29,30], suggesting that tumor formation can be initiated by the activation of an oncogene by retrotransposition or by the inactivation of oncosuppressors [31,32]. Insertion of full-length SINE-VNTR-Alu retrotransposons (SVT) into intron 8 of the caspase 8 gene (CASP8) is associated with an increased risk of basal cell carcinoma and breast cancer, but reduces the risk of prostate cancer [33]. An increase in the abundance of SINE and LINE elements in blood cfDNA at the development of melanoma B16, lung carcinoma LLC and drug resistant lymphosarcoma RLS_40_ correlated with the number of metastases, and in response to treatment, a decrease in the number of metastases was accompanied by a proportional decrease in the representation of SINE and LINE elements [20]. The potential participation of such DNAs in metastases development poses an urgent task of developing and researching DNase-based antimetastatic and antitumor drugs.

Pulmozyme^®^ is a commercially available drug that is licensed as an expectorant and mucolytic agent in an inhalation formulation for the treatment of rare respiratory diseases. The pharmacological action of Pulmozyme^®^ results in the degradation of cfDNA of purulent discharge, released from decaying leukocytes, which accumulates in lungs as a response to infection, facilitating discharge colliquation. Considering the clinical efficacy of the drug in destroying DNA, it seemed reasonable to evaluate its effect on metastasis development in mice models with lung metastases.

In this work, we studied the antimetastatic potential of Pulmozyme^®^ in a murine B16 melanoma model with intramuscular and intranasal administration and searched for correlations between levels of metastasis development, DNase activity, cfDNA concentrations and levels of c-Myc fragments and tandem repeats of SINE and LINE elements in the blood serum. The ability of Pulmozyme^®^ and DNase I to affect tumor cells in metastatic foci and cfDNA involved in the formation of NETs was also studied.

## 2. Results

### 2.1. Comparison of DNase Activity of Pulmozyme^®^ and DNase I

The antitumor and antimetastatic activity of bovine pancreatic DNase I was well investigated by our group. In particular, we identified concentrations and doses of DNase I at which the inhibition of migration of B16 melanoma cells *in vitro* and metastasis development in the B16 *in vivo* model were observed [14,20]. We should underline that it is the enzymatic activity of DNase I that determines its antitumor and antimetastatic effects. Therefore, initially, the DNase activity of Pulmozyme^®^ was compared with that of DNase I to adjust the specific activity of the enzyme and choose concentrations and doses of Pulmozyme^®^ similar to those of DNase I used for its most pronounced effect, both *in vitro* and *in vivo*.

DNase activity of both enzymes was compared by measuring the cleavage extent of pMDR670 plasmid DNA (Figure S1, Supplementary materials). Pulmozyme^®^ or DNase I (0.001–0.1 U) were incubated with plasmid pMDR670 at 37 °C for 15 min, and the cleavage products were analyzed by electrophoresis in a 1% agarose gel. The cleavage extent was calculated as the ratio of the sum of linearized and relaxed plasmid forms to the sum of linearized, relaxed and supercoiled plasmid forms (see Materials and Methods).

As depicted in Figure S1, the concentration dependencies of pMDR670 cleavage extent by Pulmozyme^®^ and DNase I are very similar, thus showing that Pulmozyme^®^, the pharmaceutical composition containing recombinant human DNase I, has the same specific activity as bovine pancreatic DNase I. The enzyme can be used at similar concentrations and doses: 2000 Kunitz/mg of bovine DNase I is equal to 1000 U/mg of Pulmozyme^®^.

### 2.2. The Effect of Pulmozyme^®^ on B16 Cell Proliferation, Apoptosis and Migration In Vitro

**Figure 1:**
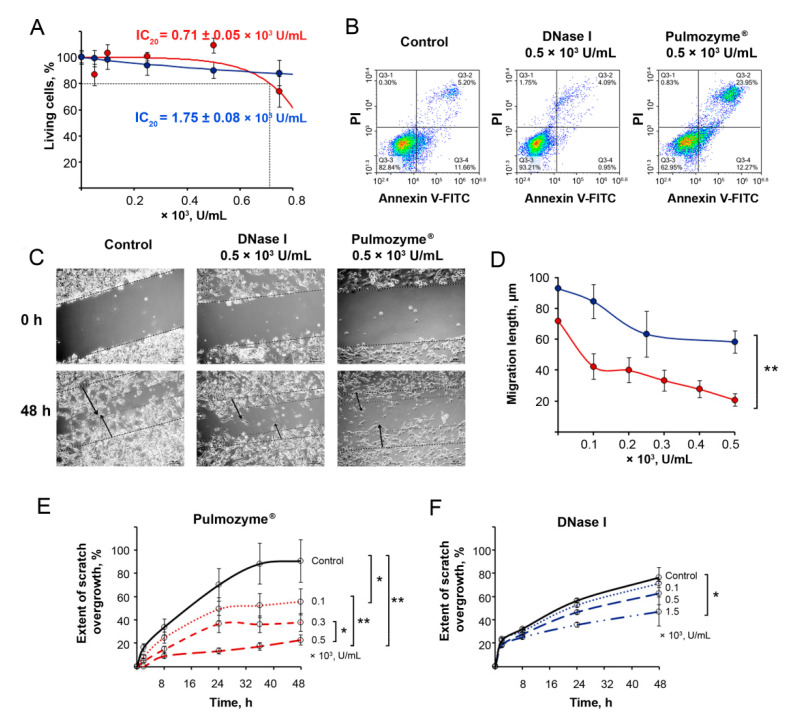
The effect of Pulmozyme^®^ and DNase I on the viability, apoptosis and migration of melanoma B16 cells. (**A**) Viability of B16 melanoma cells in presence of Pulmozyme^®^ or DNase I ((0.1–0.75) × 10^3^ U/mL) for 48 h. Number of living cells in the control (cells incubated in absence of enzyme) was taken as 100%. Data are presented as mean ± SEM. (**B**) Apoptosis of B16 cells after treatment with Pulmozyme^®^ and DNase I (0.5 × 10^3^ U/mL). Flow cytometry analysis of B16 cells stained with Annexin V-FITC/PI 24 h after treatment is shown. Q3-1, Annexin V-FITC^−^/PI^+^, necrosis; Q3-2, Annexin V-FITC^+^/PI^+^, late apoptosis; Q3-3, Annexin V-FITC^−^/PI^−^ cells; Q3-4, Annexin V-FITC^+^/PI^−^ early apoptosis. (**C**) Photographs of scratch healing by B16 cells incubated with Pulmozyme^®^ or with DNase I (0.5 × 10^3^ U/mL) (4× magnification). Dotted line shows initial edge of the scratch at time point 0; double dotted lines show boundary of the monolayer at time point 48 h. Arrows show the movement of the migration front. (**D**) Dependencies of migration length of B16 cells on Pulmozyme^®^ (red) or DNase I (blue) concentration. Data are presented for 48 h of incubation. (**E**,**F**) Extent of scratch overgrowth of B16 cells incubated with various concentrations of Pulmozyme^®^ (**D**) or DNase I (**E**). Migration of intact cells (control)—black curve. * and **—statistically significant differences with *p* < 0.05 and *p* < 0.01.

The effect of Pulmozyme^®^ and DNase I on the viability of B16 melanoma cells is shown in Figure 1A. Pulmozyme^®^ in the concentration range of 0.1–0.5 × 10^3^ U/mL only slightly decreases the viability of B16 cells, which amounted to 90–100%. With the increase in the Pulmozyme^®^ concentration to 0.75 × 10^3^ U/mL, the cell viability decreased by only 20%, and a further increase in the concentration did not affect cell viability. So, for Pulmozyme^®^ only IC_20_ was reached: IC_20_ = (0.71 ± 0.05) × 10^3^ U/mL (Figure 1A). For DNase I, the IC_20_ was three times higher compared to Pulmozyme^®^: IC_20_ = (1.75 ± 0.08) × 10^3^ U/mL (Figure 1A). Both Pulmozyme^®^ and DNase I essentially decreased the concentration of cfDNA bound to the cell surface and in the culture medium (Table S1, Supplementary materials).

The ability of Pulmozyme^®^ and DNase I to induce apoptosis of B16 melanoma cells was investigated and depicted in Figure 1B. Pulmozyme^®^ in the concentration of 0.5 × 10^3^ U/mL induced apoptosis in 30–40% of cells for 24 h (10–15% of cells were detected in early apoptosis, 15–25% in late apoptosis), despite the fact that the toxic effect of Pulmozyme^®^ at the same concentrations was insignificant. The data showed that DNase I did not induce apoptosis of B16 cells under the same conditions (Figure 1B).

The effect of Pulmozyme^®^ on the migration activity of B16 melanoma cells was investigated using the scratch assay. The integrity of the monolayer formed by the cells was violated by scratching and the rate of its filling with tumor cells was monitored in the absence (control) and presence of Pulmozyme^®^ at concentrations of (0.1–0.5) × 10^3^ U/mL or DNase I at concentrations of (0.1–1.5) × 10^3^ U/mL (Figure 1C–F). In these experiments, DNase I was used as a positive control (Figure 1C–F).

In the control after 48 h of incubation, almost the entire scratch was filled with cells (Figure 1C). At the same time, DNase I (0.5 × 10^3^ U/mL) significantly inhibited cell motility and the scratch area was only filled to 40%; Pulmozyme^®^ used at the same concentration caused stronger inhibition of cell motility and the scratch was filled no more than 20% (Figure 1C). Furthermore, cells migrated in dense groups in the control well, while cells migrated in more rarefied groups after DNase I treatment; after Pulmozyme^®^ treatment, the migration of scattered cells forming fewer contacts with each other was observed (Figure 1C). Obviously, the latter is a consequence of a decrease in the adhesive ability of the cells. Since cells do not divide in FBS-free medium, the observed migration in dense groups reflects their adhesive potential.

The obtained data clearly show that Pulmozyme^®^, similar to DNase I, decreased the migration of B16 cells in a concentration-dependent manner: Pulmozyme^®^ at the concentration of 0.1 × 10^3^ U/mL caused a 2-fold decrease in migration length, and an increase in its concentration to 0.5 × 10^3^ U/mL resulted in the 4.3-fold suppression of cell migration (Figure 1D). In the case of DNase I, the effect on the cell motility was lower: DNase I at a concentration of 0.5 × 10^3^ U/mL resulted in a 1.6-fold decrease of cell migration (Figure 1D). Previously, a 3- to 5-fold retardation of cell migration was found for DNase I used at higher doses [14]; however, due to production limitations (concentration of dornase alfa in Pulmozyme^®^), we only studied bovine DNase I in this concentration range.

Detailed analysis of the scratch healing rate shown in Figure 1E,F showed that the decrease in cell migration rate in the Pulmozyme^®^-treated wells took place starting 8 h after scratching for all concentration ranges and reached a plateau at 24 h of incubation. At longer incubations, no changes in cell migration were observed (Figure 1E). At the concentration of 0.1 × 10^3^ U/mL, Pulmozyme^®^ caused a 2-fold reduction in migration rate (24 h time point) and entirely blocked cell migration at the concentration of 0.5 × 10^3^ U/mL, while at the same time being non-toxic for the cells (Figure 1E). DNase I demonstrated the less effective inhibition of cell migration (Figure 1F): the enzyme only decreased the migration of B16 cells by 1.4- to 2-fold, even at a concentration of 1.5 × 10^3^ U/mL (Figure 1F). Thus, while being non-toxic for cells, Pulmozyme^®^ more effectively reduced B16 cell migration *in vitro* compared to DNase I at similar concentrations.

### 2.3. The Effect of Intramuscular (i.m.) Administration of Pulmozyme^®^ on the Development of Metastases In Vivo

To assess the effect of Pulmozyme^®^ on the tumor invasive potential, the metastatic model of B16 melanoma was used. In this model, B16 melanoma cells implanted intravenously did not form a primary tumor node but effectively generated metastases in the lungs and liver. DNase I serves as a positive control of antimetastatic action; for this reason, only the DNase I dose, which was the most effective in the B16 melanoma model (50 U/mouse, corresponds to 1.25 mg/kg), was used [20]. It should be noted that DNase I exhibited antitumor and antimetastatic activity when administered intramuscularly, as shown previously [13,14,19,20]. However, since Pulmozyme^®^, containing human recombinant DNase I, is a pharmaceutical composition intended for inhaled administration, in this study, we used two types of administration: intramuscular (i.m.) and intranasal (i.n.). Moreover, since the main organs for melanoma metastasizing are the lungs, we considered this model to be the most suitable to study the antimetastatic potential of Pulmozyme^®^ administered intranasally.

#### 2.3.1. Scheme of the Intramuscular Experiment

**Figure 2:**
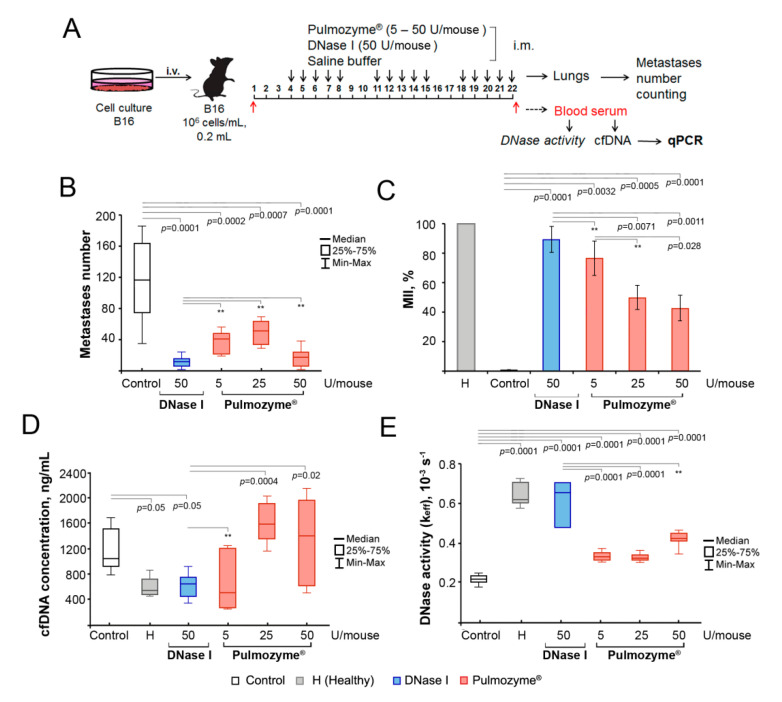
The effect of Pulmozyme^®^ and DNase I at intramuscular administration on metastases development, DNase activity and concentration of cfDNA in blood serum. (**A**) Scheme of the animal experiment. B16 cells (5 × 10^5^ cells/mL, 0.2 mL) were implanted intravenously (i.v.) into C57Bl mice (male). Starting from day 4, animals received i.m. saline buffer, Pulmozyme^®^ (5, 25 or 50 U/mouse) or DNase I (50 U/mouse) daily, except for the weekend (5 + 2). On day 22, 1 h after the last administration, blood sampling was performed, then animals were euthanized and the lungs were collected. cfDNA was isolated from blood serum and used for subsequent qPCR analysis. (**B**) Number of surface lung metastases. (**C**) Internal metastases. MII = ([mean metastasis area_control_—mean metastasis area_experiment_]/mean metastasis area_control_) × 100%. Data are presented as mean ± SE. (**D**,**F**) Concentration of cfDNA (**D**) and DNase activity (**E**) of blood serum. Data are presented as median. Data were statistically analyzed using one-way ANOVA with a post hoc Tukey test. Statistical significance *p* < 0.05. **—statistically insignificant difference. Control group—columns/boxes outlined in black, H (healthy)—colored in grey, DNase I—blue, Pulmozyme^®^—red.

B16 cells were implanted i.v.; starting from the fourth day, mice were treated i.m. by the saline buffer (control), DNase I (1.25 mg/kg, or 50 U/mouse), or Pulmozyme^®^ (0.25–2.5 mg/kg, or 5–50 U/mouse) according to the 5 + 2 scheme (Figure 2A). At the end of the experiment, we evaluated the number of surface metastases in the lungs of B16-bearing mice, as well as the areas of internal metastases in the lungs, with subsequent calculation of the metastasis inhibition index (MII; for details, see Materials and Methods). The peripheral blood of experimental animals was collected 1 h after the last administration of DNase I or Pulmozyme^®^ and used for the evaluation of DNase activity, cfDNA concentration and the levels of SINE and LINE repeats and c-Myc gene fragments by qPCR.

#### 2.3.2. Surface and Internal Metastases

Pulmozyme^®^ via i.m. administration caused a significant reduction in the number of surface lung metastases in B16-bearing mice (Figure 2B): a 2-fold reduction of metastases number (median 41 ± 6 and 51 ± 8, respectively, vs. 116 ± 19 in control) at the doses of 5 and 25 U/mouse and a 10-fold reduction (median 17.5 ± 4 vs. 116 ± 19 in control) at the dose of 50 U/mouse (Figure 2B). DNase I at the dose of 50 U/mouse caused a 10-fold decrease in the number of surface lung metastases (median 12 ± 3 vs. 116 ± 19 in control, Figure 2B). Thus, Pulmozyme^®^ and DNase I administered intramuscularly at the dose of 50 U/mouse exhibited similar effects on the development of surface metastases. Only a few liver metastases were found in the control group without treatment, and liver metastases were absent in the DNase and Pulmozyme^®^ groups (primary data not shown).

The inhibition of internal lung metastases was assessed by calculating the average area occupied by metastasis in the target organs and the metastatic inhibition index (MII) using morphometric analysis (see Materials and Methods). The MII in the control group was set at 0%, and the MII indicating the absence of metastases was set at 100%. The MII values in the groups of B16-melanoma-bearing mice that received i.m. Pulmozyme^®^ or DNase I are shown in Figure 2C.

The MII in the groups of animals that received i.m. Pulmozyme^®^ was 77 ± 5%, 50 ± 5% and 43 ± 11% for the doses of 5, 25 and 50 U/mouse, respectively, thus showing that with the increase in Pulmozyme^®^ dose, its action on internal metastasis dropped (Figure 2C). It is clear that Pulmozyme^®^ more efficiently inhibited the development of surface lungs metastasis, but failed to inhibit the formation of internal metastases. It is likely that the inability of Pulmozyme^®^ to inhibit internal metastasis resulted from the low systemic absorption when administered intramuscularly [6]. DNase I (50 U/mouse) effectively inhibited internal metastasis development: the MII reached 89 ± 3% (Figure 2C).

#### 2.3.3. cfDNA and DNase Activity

The cfDNA levels in the blood serum of healthy and tumor-bearing mice and those after the i.m. treatment with Pulmozyme^®^ or DNase I are shown in Figure 2D. The concentration of cfDNA in the blood of healthy C57Bl mice was 630 ± 94 ng/mL (Figure 2D, Table S2, Supplementary materials). B16 progression (control) resulted in a 2-fold increase in cfDNA concentration and reached a median of 1191 ± 87 ng/mL. Pulmozyme^®^ administered i.m. at the dose of 5 U/mouse caused a significant reduction in cfDNA level in the blood serum of mice with B16 (reached 724.5 ± 182 ng/mL), but at higher doses (10 and 50 U/mouse), this effect expired (1670 ± 124 and 1463 ± 303 ng/mL, respectively, Figure 2D, Table S2). DNase I (50 U/mouse) administered i.m. caused a reduction of cfDNA level up to 676 ± 70 ng/mL, which is close to the cfDNA level in healthy mice (Figure 2D, Table S2).

The DNase activity in the blood serum of healthy mice, calculated as k_eff_, was (0.62 ± 0.04) × 10^−3^s^−1^ (Figure 2E, Table S3, Supplementary materials). With the progression of B16 melanoma, a 3-fold decrease in DNase activity was noted: k_eff_ = (0.22 ± 0.008) × 10^−3^s^−1^. Pulmozyme^®^ at the doses used (5, 10 and 50 U/mouse) demonstrated only a tendency towards an increase in the DNase activity of blood plasma, reaching (0.33 ± 0.01) × 10^−3^s^−1^, (0.32 ± 0.01) × 10^−3^s^−1^ and (0.43 ± 0.02) × 10^−3^s^−1^, respectively. The i.m. injections of DNase I (50 U/mouse) led to a significant increase in the DNase activity of blood serum and restored DNase activity to the level of healthy animals (Figure 2E, Table S3).

Apparently, Pulmozyme^®^ administered i.m., even at the highest dose, caused an insignificant reduction of cfDNA level, which correlated well with the insufficient restoration of serum DNase activity and with its moderate effect on internal metastases formation.

### 2.4. The Effect of the Intranasal (i.n.) Administration of Pulmozyme^®^ on Metastasis Development In Vivo

#### 2.4.1. Scheme of the Intranasal Experiment

**Figure 3:**
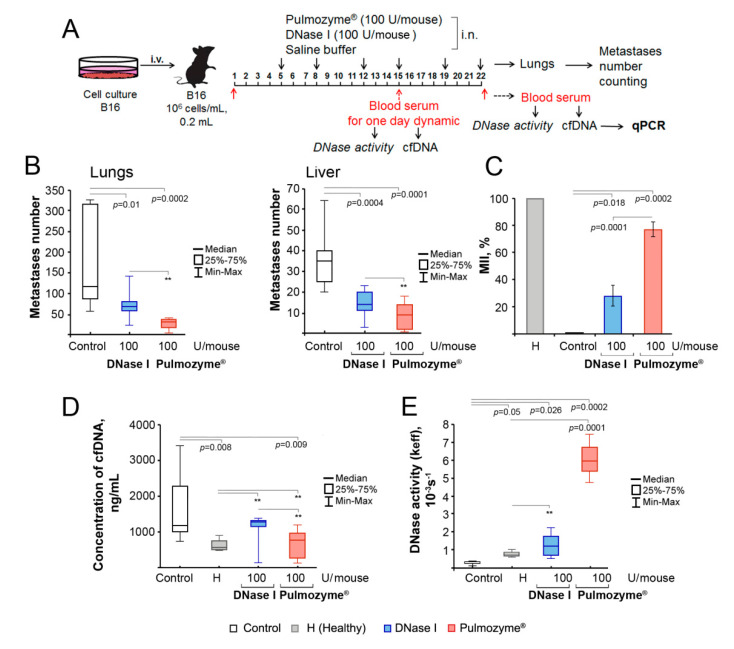
The effect of Pulmozyme^®^ and DNase I administered intranasally on metastases development, DNase activity and concentration of cfDNA in blood serum of mice with B16. (**A**) Scheme of the animal experiment. B16 cells (5 × 10^5^ cells/mL, 0.2 mL) were implanted intravenously (i.v.) into C57Bl mice (male). Starting from day 5, animals received i.n. saline buffer, Pulmozyme^®^ (100 U/mouse) or DNase I (100 U/mouse) twice a week (six administrations). On days 15 and 22, 1 h after administration, blood sampling was performed. On day 22 after blood sampling, animals were euthanized and the lungs and liver were removed. cfDNA was isolated from blood serum and used for subsequent qPCR analysis. (**B**) Number of surface lung (left panel) and liver (right panel) metastases, respectively. (**C**) Metastasis inhibition index (MII) in lungs. Data are presented as mean ± SE. (**D**), (**E**) cfDNA and DNase activity of blood serum of mice with B16, respectively. Data are presented as median. Data were statistically analyzed using one-way ANOVA with a post hoc Tukey test. Statistical significance *p* < 0.05. **—statistically insignificant difference. Control group—columns/boxes outlined in black, H (healthy)—colored in grey, DNase I—blue, Pulmozyme^®^—red.

B16 cells were implanted i.v.; starting from the fifth day, mice were treated i.n. by saline buffer (control), DNase I (2.5 mg/kg, or 100 U/mouse) or Pulmozyme^®^ (5 mg/kg, or 100 U/mouse) two times per week on 5, 8, 12, 15, 19 and 22 days after tumor implantation (Figure 3A). To estimate the antimetastatic efficacy of Pulmozyme^®^, we had to use this dose as the maximum possible, taking into account the fact that the volume of intranasal administration cannot exceed 100 μL.

#### 2.4.2. Surface and Internal Metastases

At the end of the experiment, the number of surface lung and liver metastases of B16-bearing mice was evaluated, along with the areas occupied by internal metastasis in the lungs. Blood samples collected as described above were used for the evaluation of DNase activity, cfDNA concentration and the levels of SINE and LINE repeats and Myc gene by qPCR.

The intranasal administration of both Pulmozyme^®^ and DNase I resulted in a significant reduction in the number of surface lung and liver metastases in tumor-bearing mice (Figure 3B). Pulmozyme^®^ (100 U/mouse) caused a 4-fold decrease in the number of lung and liver metastases (median 30.5 ± 4 vs. 115 ± 50 in the control and 9 ± 2 vs. 35 ± 6 in the control, respectively). DNase I at the same administered dose (100 U/mouse) was less effective and caused a 2- to 2.5-fold decrease in the number of lung and liver metastases (median 66.5 ± 14 vs. 115 ± 50 in the control and 14 ± 3 vs. 35 ± 6 in the control, respectively) (Figure 3B). However, the differences between Pulmozyme^®^ and DNase I groups were statistically insignificant.

Pulmozyme^®^, via i.n. administration, exhibited high efficiency in the inhibition of the development of internal lung metastases: MII reached 72 ± 6% and was much higher than MII of DNase I, which was 38 ± 9%. It is worth mentioning that the i.n. administration of Pulmozyme^®^ was more effective than DNase I, even though the doses of the enzymes were twice as high (Figure 2C and Figure 3C).

#### 2.4.3. cfDNA and DNase Activity

In mice treated i.n. with saline buffer (control), the cfDNA concentration in the blood serum reached 1180 ± 343 ng/mL, which was 2-fold higher compared to that in healthy animals (567 ± 94 ng/mL, Figure 3D, Table S2, Supplementary materials). Pulmozyme^®^ administered i.n. caused a 1.5-fold decrease in cfDNA concentration, which was close to the level of healthy animals (765 ± 122 ng/mL, Figure 3D, Table S2). DNase I via i.n. administration did not change cfDNA concentration (reached 1285 ± 152 ng/mL, Figure 3D, Table S3, Supplementary materials). Despite the fact that Pulmozyme^®^ more effectively decreased cfDNA concentration than DNase I, there were no statistically significant differences between these groups.

B16 progression in the control group was accompanied by a 3-fold decrease in the DNase activity of blood serum compared to healthy mice ((0.26 ± 0.03) × 10^−3^ s^−1^ vs. (0.62 ± 0.04) × 10^−3^ s^−1^, respectively). The intranasal administration of Pulmozyme^®^ caused a 23-fold increase in the DNase activity of blood serum compared to the control, whereas DNase I administration increased DNase activity only 5-fold: k_eff_ reached (5.95 ± 0.24) × 10^−3^s^−1^ for Pulmozyme^®^ and (1.20 ± 0.22) × 10^−3^s^−1^ for DNase I (Figure 3E, Table S3).

Thus, it is obvious that the antimetastatic activity of Pulmozyme^®^ and DNase I, as well as cfDNA/DNase activity of blood serum, depends on the type of administration. Pulmozyme^®^ is substantially more effective via intranasal administration, while DNase I was more effective via intramuscular administration.

### 2.5. Histological Structure of Metastatic Foci in the Lungs of Mice with B16 after i.m. and i.n. Administration of Pulmozyme^®^ and DNase I

**Figure 4:**
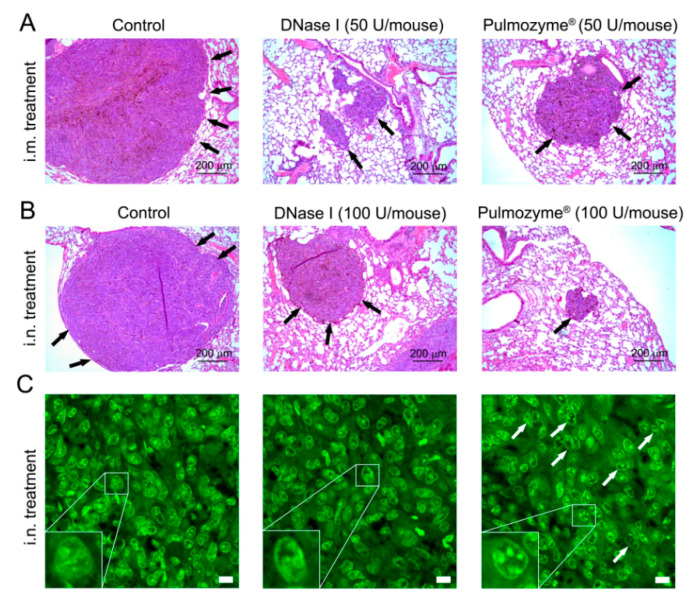
Representative images of B16 melanoma metastases in the lungs of tumor-bearing mice without treatment (control) and after i.m. and i.n. administration of Pulmozyme^®^ and DNase I. (**A**,**B**) Metastases in lungs in intramuscular (**A**) and intranasal (**B**) experiments. Hematoxylin and eosin staining, light microscopy, original magnification ×100. Black arrows indicate metastatic foci in the lungs. (**C**) SYTO^TM^ staining, confocal fluorescent microscopy using a plan-apochromat 63×/1.40 Oil DIC M27 objective. Scale bar corresponds to 10 μm. White arrows indicate B16 melanoma cells with disrupted structural organization of nuclei. Typical examples of B16 cells with condensation and parietal localization of chromatin are shown in the bottom left corner.

Histological analysis revealed that the metastatic foci of B16 melanoma in the lungs of tumor-bearing mice had a similar structure in both the experimental and control groups—rounded shape with clear boundaries and located predominantly around the bronchi and blood vessels (Figure 4A,B). Representative images of lung sections depicted in Figure 4 clearly demonstrate that the i.m. administration of DNase I led to a more significant reduction in internal metastasis development than the i.m. administration of Pulmozyme^®^, whereas i.n. administration was more effective in the case of Pulmozyme^®^, which was quantified by MII counting (Figure 2, Figure 3 and Figure 4A,B). Histologically, metastatic foci of B16 melanoma in the lungs of tumor-bearing mice of all groups were represented by polymorphic atypical cells containing the brown pigment melanin (Figure 4A,B).

To prove or refute the possible effects of DNases on NETs, we tried to visualize cfDNA in NET structures by SYTO^TM^ staining and the products of neutrophils after NET-osis by staining with mAb against neutrophils (Ly-6B antigen) and against neutrophil elastase. For DNA visualization, SYTO^TM^ staining of histological sections of lungs with metastases from mice with B16 treated i.n. with saline buffer, Pulmozyme^®^ or DNase I was performed, and the structural organization of the nucleus of tumor cells was analyzed (Figure 4C and Figure S2).

An interesting observation was made that Pulmozyme^®^ and DNase I treatment led to the violation of nuclear morphology and disruption of the structural organization of the B16 cells’ chromatin: the nuclei with internal voids were disintegrated and wrinkled and intercellular spaces were expanded (Figure 4C). Moreover, nuclear chromatin condensation and fragmentation with the formation of nuclear vacuoles and a large number of polymorphic granules and parietal localization of chromatin close to the nuclear membrane were observed, which characterizes the degradation of nucleosomal DNA, and DNA packing in microparticles, indicating cell death via necrosis or late-stage apoptosis, which is consistent with data in the literature [34,35,36]. The detected changes were more pronounced with the i.n. administration of Pulmozyme^®^ (Figure 4C). Most of the B16 cells in the control group without treatment remained uniform and intact, the cavities inside the nucleus were smaller and most nuclei had normal morphology, while the cells demonstrated no considerable sign of apoptosis (Figure 4C). It should be noted that due to strong signals of nuclear chromatin in the metastatic foci in the lung samples, cfDNA was not detected in the intercellular space, neither in the control group or in the Pulmozyme^®^ or DNase I groups by SYTO^TM^ staining. 

The effect of DNase I and Pulmozyme^®^ on apoptosis induction was evaluated by fluorescence-based immunohistochemistry with mAb against caspase-3 and caspase-7 (Figures S2A,B, S3 and S4). The intranasal administration of Pulmozyme^®^ (100 U/mouse) to B16 melanoma-bearing mice did not change the expression of caspase-3 and enhanced the expression of caspase-7 by 21% in lung metastasis foci compared to the control group (Figures S2A,B, S3 and S4). Intranasal administration of DNase I at the same dose (100 U/mouse) did not affect expression of apoptosis-associated markers. Thus, our hypothesis was confirmed that the change in the chromatin structural organization in the nuclei of metastatic foci after Pulmozyme^®^ administration is the manifested sign of the late-stage apoptosis.

The effect of DNase I and Pulmozyme^®^ on the neutrophil recruitment in the metastasis foci and expression of neutrophil elastase was evaluated (Figures S2C,D, S5 and S6). The intranasal treatment of tumor-bearing mice with DNase I and Pulmozyme^®^ (100 U/mouse for both) led to 5.1- and 6.9-fold increases in fluorescent signal in the metastatic foci in comparison with the control animals (Figures S2C and S5). However, since cells in the structure of the lungs include inflammatory monocytes and some activated macrophages, as well as lung tissue containing the same determinants (Ly-6B) as neutrophils, staining with mAb to neutrophils did not allow the question about neutrophil recruitment to be answered.

However, the effect of DNase I and Pulmozyme^®^ on NETs made it possible to elucidate neutrophil elastase (Figure S2D). The intranasal treatment of tumor-bearing mice with DNase I and Pulmozyme^®^ led to decreases of 63.3% and 59.6% in level of neutrophil elastase, respectively, in the metastatic foci in comparison with the control animals (Figures S2D and S6). Decrease in elastase level indicates disaggregation of NETs after DNase I and Pulmozyme^®^ administration.

### 2.6. One-Day Dynamic of cfDNA Concentration and DNase Activity in Blood Serum of Mice with B16 after i.n. Administration of Pulmozyme^®^ and DNase I

Since the best antimetastatic effect was achieved for Pulmozyme^®^ after intranasal administration, we analyzed the one-day dynamic of cfDNA concentration and DNase activity in the blood serum of mice with B16 after the i.n. administration of Pulmozyme^®^ and DNase I. The study was carried out on the 15th day of the experiment after three administrations of enzymes, suggesting their pronounced effect on concentration and activity to that time point.

**Figure 5:**
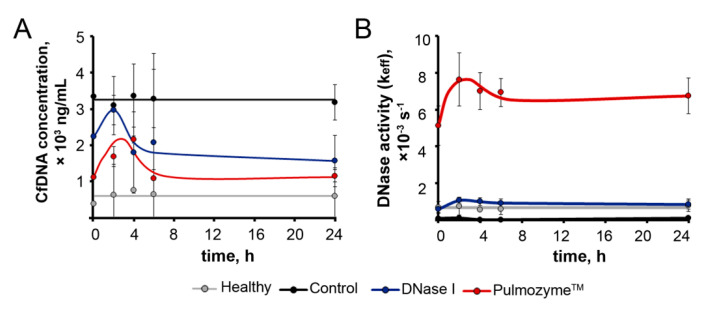
The one-day dynamic of cfDNA concentration and DNase activity in blood serum of mice with metastatic B16 after the i.n. treatment with Pulmozyme^®^ or DNase I: summary data. (**A**) cfDNA concentration. (**B**) DNase activity. Treatment scheme is presented in Figure 3A. On day 15 after B16 implantation, the fourth i.n. administration of saline buffer (control), Pulmozyme^®^ and DNase I to mice with B16 was performed; after 0, 2, 4, 6 and 24 h, blood samples were collected, and cfDNA and DNase activity in blood serum was measured. Data are presented as mean ± SE. Healthy group is marked in grey, control group—black, DNase I group—blue, Pulmozyme^®^ group—red.

On day 15, after B16 implantation and the fourth i.n. administration of saline buffer (control, Figure 3A), Pulmozyme^®^ and DNase I blood sampling was performed at 0, 2, 4, 6 and 24 h time points. Healthy mice were used in parallel for this type of analysis to control the effect of blood sampling on cfDNA level and DNase activity (Figure 5). Figure 5 shows the averaged data from several animals; Figures S7 and S8 show individual dynamics plots for each animal (Supplementary Materials).

Kinetics of cfDNA in all groups, with the exception of the control, were characterized by a rise in cfDNA level for 2–6 h (Figure S7A,C,D). In the control group, the increase in cfDNA level was observed in two of the three mice (Figure S3B). In the case of the Pulmozyme^®^ and DNase I groups, a gradual decline in cfDNA concentrations was observed to levels lower than the initial cfDNA level at time point 0 within 24 h (Figure S7A–D). It should be noted that the cfDNA concentration in mice that did not drop out of the experiment did not decrease lower than the initial cfDNA concentration at time point 0, even on the 22nd day (end of the experiment) (Figure S7A–D). A slight increase in cfDNA concentration in the healthy group indicates the effect of blood sampling due to a violation of tissue integrity.

The data on DNase activity presented in Figure S8 clearly demonstrate that a strong increase in the DNase activity of blood serum for 2–6 h was observed in the DNase I and Pulmozyme^®^ groups in comparison with initial levels (Figure S8C,D). Furthermore, in the DNase I group, a gradual decline in DNase activity was demonstrated, which, nevertheless, did not drop below the initial level (Figure S8C). In the Pulmozyme^®^ group, only a slight decrease in DNase activity took place within 24 h, which exceeded the initial level more significantly (Figure S8D). In healthy animals, we found weak DNase activity fluctuations that could be explained by both intranasal administration and frequent blood sampling (Figure S8A).

We also analyzed the resulting curves, which are superpositions of curves for all animals in the group (Figure 5). The concentration at time point 0 h was taken as a baseline level: 389 ± 117 ng/mL for healthy animals, 3348 ± 116 ng/mL for the control group, 2232 ± 757 ng/mL and 1124 ± 377 ng/mL for mice with B16, which received DNase I and Pulmozyme^®^, respectively. Comparison of the baseline levels revealed that the level of cfDNA in the control group was 8.5-fold higher than the level of cfDNA in healthy animals, whereas this level was 1.5- and 3-fold lower, respectively, in the DNase I and Pulmozyme^®^ groups (Figure 5A). These differences in baseline levels in the Pulmozyme^®^ and DNase I groups likely resulted from the effectiveness of enzyme action during 10 days of treatment.

In control mice, after the saline administration and further blood sampling, concentrations of cfDNA insignificantly decreased over 2 h, and the mean cfDNA level was restored to baseline (Figure 5A). In the DNase I group after DNase I administration and blood sampling, we observed a 1.3-fold increase in cfDNA concentration, with a subsequent decrease to the level a little below baseline, while we observed a 2-fold increase in cfDNA level over 2 h in the Pulmozyme^®^ group, with a subsequent decrease that was not lower than the baseline level (Figure 5A).

DNase activity in the blood serum of mice 1 h after intranasal administration and just after the first blood sampling (0 h) was taken as a baseline level: (0.70 ± 0.3) × 10^−3^s^−1^ for healthy animals, (0.09 ± 0.02) × 10^−3^s^−1^ for the control group, (5.1 ± 1.0) × 10^−3^s^−1^ for Pulmozyme^®^ and (0.59 ± 0.2) × 10^−3^s^−1^ for DNase I groups. It can be clearly seen that DNase activity in the control group was 7 times lower than in the group of healthy mice (black and grey curves, respectively, Figure 5B). In the DNase I group, we observed a 6.5-fold increase in the DNase activity of blood serum over 2–6 h relative to the control group after DNase I administration. In the Pulmozyme^®^ group, the most substantial increase in serum DNase activity of 57 times was observed during 2–6 h when compared to the control group (Figure 5B).

In the DNase I group, in the first 2 h after sampling, a 2-fold increase in DNase activity was observed with a subsequent decline close to, but not lower than, the baseline level. In the Pulmozyme^®^ group within 2 h after sampling, a 1.5-fold increase in DNase activity was observed followed by a slight decrease in activity within 6 h, after which the activity remained at the same level, twice the baseline (Figure 5B).

It should be noted that Pulmozyme^®^ generated DNase activity in the blood serum that was 10 times higher than that of DNase I. The obtained data clearly show that after the administration of Pulmozyme^®^, the level of DNase activity was maintained for 24 h and enhanced the baseline level 2 times at the 24 h time point (Figure 5B and Figure S8C).

### 2.7. Analysis of the Abundance of SINE and LINE Elements and the c-Myc Gene in the Blood of Mice with B16

Earlier, we demonstrated that the antimetastatic action of DNase I in a model of Lewis lung carcinoma correlated with a decrease in the total level of tandem repeats (next-generation sequencing data) [19]. Using three murine tumor models, including melanoma B16, it was confirmed that the antimetastatic effect of DNase I correlated with the decrease in the levels of tandem repeats of SINE/LINE elements and some oncogenes in cfDNA [20]; thus, it was proposed that SINEs and LINEs, as well as fragments of oncogenes in the cfDNA, could be the targets of DNase I in the implementation of its antimetastatic effect.

**Figure 6:**
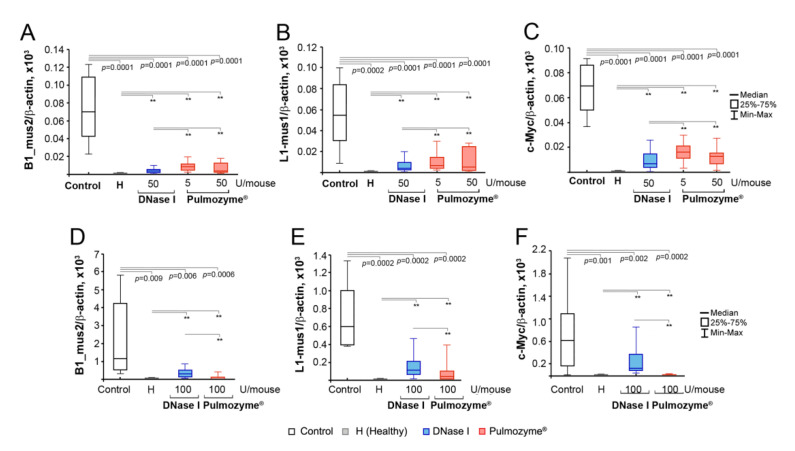
The levels of SINE element B1_mus2 (**A**,**D**), LINE element L1_mus1 (**B**,**E**) and c-Myc gene (**C**,**F**) in cfDNA of the blood serum of mice with B16 after i.m. (**A**–**C**) or i.n. (**D**–**F**) administration of Pulmozyme^®^ or DNase I. Data of qPCR. See Figure 2A (for i.m.) and Figure 3A (for i.n.) for experimental setup. The PCR meanings were normalized to the level of β-actin and expressed as arbitrary unit (a.u.). Data were statistically analyzed using one-way ANOVA with a post hoc Tukey test. Data are presented as median. Statistical significance *p* < 0.05; **—statistically insignificant difference. Control group—columns/boxes outlined in black, H (healthy)—colored grey, DNase I—blue, Pulmozyme^®^—red.

To detect the effect of Pulmozyme^®^ on SINE and LINE levels, we chose B1_mus2 (SINE) and L1_mus1 (LINE) elements and performed PCR analysis of the cfDNA isolated from the blood of healthy mice, control B16-bearing mice and those treated with Pulmozyme^®^ or DNase I i.m. and i.n. Normalized levels of SINEs and LINEs in the cfDNA samples are shown in Figure 6.

In the cfDNA of healthy mice, the SINE/LINE abundance was low and did not exceed 1–2 a.u. (Figure 6A,B,D,E, Tables S4 and S5, Supplementary materials). In the control group (i.m. administration), melanoma progression was accompanied by a significant increase in the abundance of SINE and LINE elements: the level of B1_mus2 became 700-fold higher compared to healthy animals, while L1_mus1 increased by 50 times (Figure 6B, Tables S4 and S5). The i.m. administration of Pulmozyme^®^ regardless of dose resulted in the essential decrease in the levels of SINEs and LINEs close to the baseline in the healthy group. Similarly, the i.m. administration of DNase I led to the decrease of SINE and LINE levels close to the level of healthy animals (Figure 6A,B, Tables S4 and S5). It is worth mentioning that the differences in SINE and LINE abundance between Pulmozyme^®^ and DNase I were statistically insignificant; however, DNase I administered i.m. decreased the levels of repeats somewhat more efficiently than Pulmozyme^®^ (Figure 6A,B).

c-Myc level in the cfDNA of the blood serum of healthy animals was as low as the SINE and LINE levels (Figure 6C,F, Table S6, Supplementary materials). During the B16 progression in the control group, c-Myc levels in cfDNA significantly increased up to 200-fold (Figure 6C, Table S6). Treatment with DNase I (i.m.) led to a 10-fold drop in c-Myc level compared to the control group under these conditions. Pulmozyme^®^ applied i.m. either at a dose of 5 or 50 U/mouse decreased the c-Myc level only up to 5-fold compared to the control group (Figure 6C, Table S6). It is worth mentioning that Pulmozyme^®^ administered i.m. decreased the level of c-Myc in cfDNA less efficiently than DNase I.

The i.n. experiment showed a very high abundance of SINEs and LINEs following B16 progression: B1_mus2 and L1_mus1 levels were 10,000 and 5000 times higher compared to the healthy group (Figure 6D,E, Tables S4 and S5). Pulmozyme^®^ (i.n.) caused a significant 40-fold and 20-fold reduction of both B1_mus2 and L1_mus1. DNase I via i.n. administration was found to be much less effective compared to Pulmozyme^®^.

Similar to the i.m. experiment, under the B16 progression in the intranasal experiment, c-Myc level was also found to be significantly increased compared to healthy animals (Figure 6, Table S6). The i.n. administration of DNase I led to a 7-fold decrease in c-Myc level. Pulmozyme^®^ administered i.n. caused a more pronounced drop in c-Myc level, and the final level was only insignificantly higher compared to healthy animals.

A significant difference in the levels of SINE and LINE elements and fragments of c-Myc in the control groups of i.m. and i.n. experiments was observed: 70 ± 14 vs. 1154 ± 753 a.u. for B1_mus2, 54 ± 11 vs. 598 ± 152 a.u. for L1_mus1 and 69 ± 7.4 vs. 738 ± 231 a.u. for c-Myc, respectively (Tables S4–S6). It should be noted that despite similar levels of cfDNA concentration in the blood serum of mice with B16 treated i.m. and i.n. with saline buffer, the abundance of SINE and LINE elements, as well as c-Myc oncogene fragments in the cfDNA, differed by one order of magnitude. The models of B16 melanoma in i.m. and i.n. experiments differed by several parameters, despite the same route of implantation and the same number of implanted B16 cells. Thus, in the intramuscular model, we observed the development of metastases mainly in the lungs, and only a few in the liver, while metastases developed both in the lungs and in the liver in the intranasal model. Besides that, the absolute area of internal metastases in the lungs in the intranasal model was much higher than in the intramuscular model. We suppose that just this difference gave us the one order of magnitude difference in the concentration of SINE and LINE elements and c-Myc oncogene in blood serum.

## 3. Discussion

In this study, the effects of Pulmozyme^®^, a pharmaceutical composition containing human recombinant DNase I, on the proliferation and cell migration *in vitro* and metastases development in a B16 melanoma model *in vivo* were investigated. We analyzed the correlations between alterations of the invasion potential of B16 melanoma *in vivo* and the concentration of circulating cfDNA and DNase activity in the blood serum of mice with B16 melanoma that received Pulmozyme^®^ either i.m. or i.n. In addition, the level of specific DNA fragments in the pool of cfDNA playing a role in carcinogenesis, such as SINE and LINE elements and the c-Myc oncogene, was determined. Pulmozyme^®^ is successfully used to treat cystic fibrosis, and attempts have been made to use Pulmozyme^®^ for the treatment of specific viral lung infections, pleural effusion, emphysema and autoimmune diseases [5,7,37]. However, Pulmozyme^®^ has never been used as an antimetastatic agent.

We found that *in vitro* Pulmozyme^®^ is not toxic for cells because only a slight inhibition of B16 cell proliferation under the action of Pulmozyme^®^ was observed (IC_20_ = ((0.75 ± 0.05) × 10^3^ U/mL). These data are consistent with a recent study showing that Pulmozyme^®^ had no toxic effect on the normal epithelial cells of the Vero kidney and peripheral blood mononuclear cells [38]. Being non-toxic for B16 cells, Pulmozyme^®^ was shown to efficiently suppress the migration of melanoma B16 cells *in vitro* at the doses much lower than even IC_20_, and the effect of Pulmozyme^®^ was 10 times higher than the effect of bovine pancreatic DNase I [14]. *In vitro* Pulmozyme^®^, unlike DNase I, induced apoptosis in B16 cells. Apparently, DNase I, in contrast to Pulmozyme^®^, could be inhibited by G-actin, which presents both inside the cell and in the culture medium [39]. At the same time, Pulmozyme^®^ and DNase I effectively decreased the concentration of cfDNA, both bound to the cell surface and cfDNA in the culture medium.

*In vivo*, the i.m. administration of Pulmozyme^®^ to B16-bearing mice caused a 10-fold reduction in the number of surface lung metastases; this effect was comparable with that of DNase I used at the same dose (50 U/mouse). Interestingly, the effectiveness of the inhibition of internal metastases development following the i.m. administration of Pulmozyme^®^ was 1.5 to 2 times lower than for DNase I, regardless of dose, within statistical confidence. It seems obvious that the bias effects of Pulmozyme^®^ on the development of surface and internal lung metastasis are linked to its insufficient penetration into the bloodstream at i.m. administration.

The intranasal administration of Pulmozyme^®^ resulted in the significant suppression of the development of both surface and internal lung and liver metastases, while DNase I via i.n. administration was less effective than Pulmozyme^®^. Thus, our data showed that Pulmozyme^®^ is more efficient in metastases inhibition when administered intranasally, while DNase I displayed pronounced antimetastatic activity when administered intramuscularly. We attribute this to the fact that via i.n. administration, Pulmozyme^®^, intended for inhalation administration, enters the respiratory tract and further into the lungs, thus working more effectively. 

It was shown that tumor and metastases progression are accompanied by a pathological increase in the concentration of cfDNA in the blood of patients, which, in turn, is often accompanied by the decrease in the DNase activity of blood serum [40,41,42,43]. Our data showed that metastasis spread in the metastatic melanoma model is accompanied by an increase in the concentration of cfDNA with the accumulation of specific fragments, such as SINE and LINE elements and fragments of the c-Myc gene, and an essential decrease in the DNase activity of blood serum [19,20]

The intramuscular and intranasal administration of Pulmozyme^®^ and DNase I both caused a decrease of the concentration of total cfDNA and specific fragments almost to the level of healthy animals. It should be noted that in the case of i.m. administration, we observed the restoration of DNase activity of blood serum to the level of healthy animals both for Pulmozyme^®^ and DNase I. Following the intranasal administration of DNase I, we observed an approximately 2-fold increase in DNase activity in comparison with the control, whereas Pulmozyme^®^ demonstrated a substantially higher (up to 10-fold) increase in DNase activity in the blood serum. As mentioned above, the aggressiveness of B16 melanoma in intramuscular and intranasal experiments was different: in the latter case, the aggressiveness was higher for unknown reasons and it manifested in organs occupied by metastases (lungs and liver in i.n. experiments) and the absolute area of internal metastases in lungs that resulted in a one order of magnitude difference in the concentration of SINE and LINE elements and fragments of the c-Myc oncogene in the blood serum of control mice (compare Figure 2B, Figure 3B and Figure 6A–C,D–F). Nevertheless, Pulmozyme^®^ via i.n. administration very efficiently fought against this aggressive melanoma and suppressed metastasis development in both organs (lungs and liver), demonstrating very high antimetastatic potential. 

**Figure 7:**
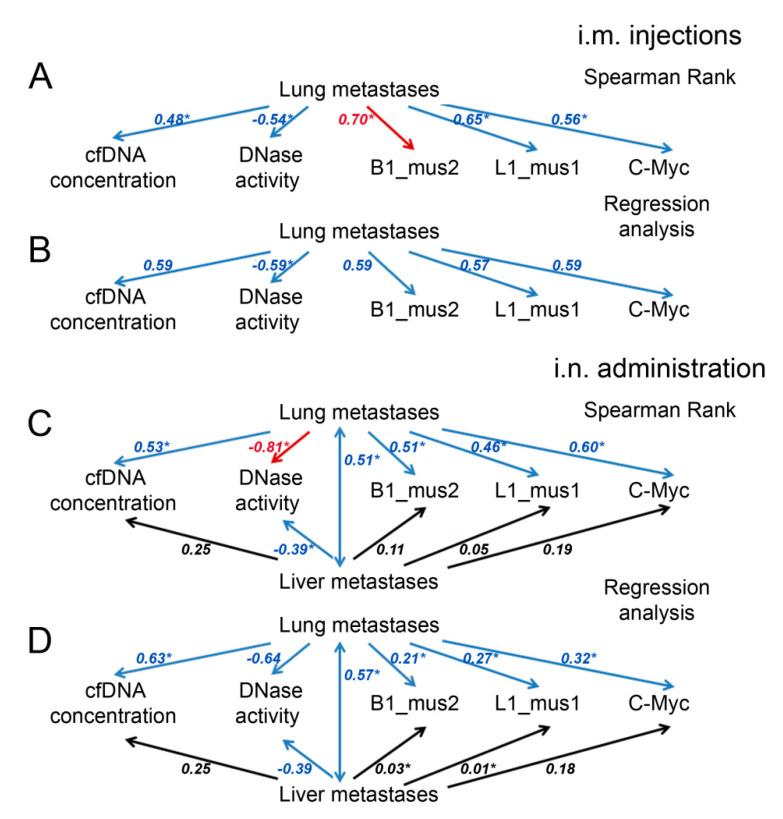
Correlations between the metastasis number, serum DNase activity, serum cfDNA concentration and B1_mus1, L1_mus1 and c-Myc levels in cfDNA. Correlation coefficients (Spearman rank, r, and regression analysis, R^2^) reflecting the statistical relationships between the studied parameters were evaluated for mice with B16 after intramuscular (**A**,**B**) or intranasal (**C**,**D**) administration of saline buffer, DNase I or Pulmozyme^®^. Red arrows indicate strong positive or negative correlations (0.70 ≤ r or R^2^ ≤ 0.99), blue arrows indicate moderate correlations (0.3 ≤ r or R^2^ < 0.7), black arrows indicate weak correlations (0.01 ≤ r or R^2^ < 0.3); * indicated statistical significance *p* < 0.05.

We analyzed the relationships between the inhibition of metastasis and different parameters, such as cfDNA concentration, DNase activity and levels of B1-mus2, L1-mus1 and c-Myc, using two statistical metrics (Spearman rank, r, and regression analysis, R^2^). A decrease in metastasis number is well correlated with a decrease in cfDNA concentration for both types of administration with *r* and R^2^ 0.5–0.6 (Figure 7, Tables S7–S12, Supplementary materials). Moreover, there were better Spearman correlations for specific fragment levels and lung metastasis number, and for B1_mus2, a strict dependence with r = 0.7 was found (Figure 7, Tables S9 and S12).

The development of melanoma is characterized by the development of lung and liver metastases. Following intranasal administration, the number of liver metastases moderately depended on the number of metastases in the lungs (r = 0.51 and R^2^ = 0.57, Figure 7, Tables S10 and S12) and weakly correlated with other parameters. Nevertheless, there was a moderate correlation between the number of liver metastases and the concentration of cfDNA (r and R^2^ = 0.25), and almost no correlations were found with the level of specific fragments (Figure 7, Tables S9 and S12). It seems likely that the lungs are the primary target for melanoma metastasis, while metastases in the liver are formed due to aggressive processes.

The correlations between the DNase activity and lung metastasis number were significantly higher compared to other parameters, and the Spearman coefficient reached r = 0.81 for i.n. administration (Figure 7, Tables S9 and S12). For liver metastasis and DNase activity, we found moderate correlations with *r* and R^2^ = 0.39 (Figure 7, Tables S9 and S12). Therefore, DNase activity appears to be the most promising parameter for assessing, firstly, the severity of the disease, and secondly, the effectiveness of treatment with Pulmozyme^®^ or DNase I.

An increased level of cfDNA often correlates with a decrease in the DNase activity of blood plasma and with treatment efficacy [44,45] However, there seems to be a more complex relationship between these parameters. Thus, increased levels of DNase activity have been found in the early stages of liver cancer and in precancerous conditions, accompanied by normal and weakly elevated levels of cfDNA [46]. It was hypothesized that an elevation in DNase activity is driven by the increased release of cfDNA and serves as a compensatory mechanism to keep cfDNA levels in check and thereby mitigate the promotion of inflammation and cancer [46].

Studying the one-day dynamic of cfDNA and DNase activity after the i.n. administration of Pulmozyme^®^ and DNase I, the most important result is that for 24 h, throughout the treatment, both Pulmozyme^®^ and DNase I maintained DNase activity in the blood serum much higher than in the control group and even in the healthy group, and Pulmozyme^®^ administered i.n. is several times more effective than DNase I. It should be noted that after four administrations of Pulmozyme^®^, the baseline DNase activity in the Pulmozyme^®^ group was much higher than in the control group, as well as the level of DNase activity on the 22nd day, which indicates the maintenance of a high level of DNase activity throughout the experiment.

To understand the mechanism of action of DNases, we tried to link the role of cfDNA with the development of metastasis, and, accordingly, the role of DNases as restraining agents relative to this. To date, two hypotheses have been put forward: metastases can occur via the transfection of vulnerable cells located in the target organs with (1) tumor-specific DNA from cells of the primary tumor circulating in the blood plasma [47] and/or (2) fragments of mobile elements and oncogenes that can behave like oncoviruses, thus opening an alternative pathway for metastasis [48,49,50].

We found that at B16 progression, not only the total concentration of cfDNA increase, but the abundance of specific elements, such as SINE and LINE elements and fragments of the c-Myc gene, were also enhanced. An increase in the level of c-Myc fragments in the pool of cfDNA with the development of Lewis lung carcinoma (LLC) and the SINE and LINE elements during the development of LLC, lymphosarcoma RLS_40_ and melanoma B16 was detected [19,20]. The reduction in the abundance of the c-Myc gene and SINE and LINE elements after treatment with Pulmozyme^®^, taking into account satisfactory correlation coefficients and a reduction in metastasis, serves as a confirmation that these sequences play a certain role in metastasis spread (Figure 7, Tables S7–S12, Supplementary materials). These data are well correlated with the similar effect of DNase I in the metastatic melanoma model [20].

Thus, DNases destroying cfDNA prevent possible malignant transformation and thereby contribute to the inhibition of metastasis development. As a result, DNases can be used as “restorers of the balance” between the cfDNA concentration and the DNase activity of blood plasma during such pathological processes as tumor progression and metastasis.

Another hypothesis about the pathological role of cfDNA in metastases spreading concerns NETs as a special cloudy network consisting of cfDNA with the neutrophil granule components integrated into this web-like structure [51]. We did not detect alterations of cfDNA level, but we detected alterations both in the neutrophil recruitment and in the level of neutrophil elastase in metastatic foci in the DNase I and Pulmozyme^®^ groups basing on which we can conclude that NETs disaggregate under the action of enzymes.

Although we cannot detect the effect of Pulmozyme^®^ and DNase I on cfDNA in NETs, we discovered that the enzymes caused the degradation of endonuclear DNA. The administration of both Pulmozyme^®^ and DNase I led to the activation of apoptosis in metastatic foci with an increase in cfDNA concentration. Apoptosis was confirmed by morphological changes in metastases in lungs, where DNase I and especially Pulmozyme^®^ caused the condensation of chromatin in the form of clumps in the nuclei, and these degenerative changes indicate cell death via necrosis or late-stage apoptosis [34,35,36,52].

Thus, summarizing all of the above, it should be noted that DNases combine several mechanisms, giving an impact to the antimetastatic effect: (i) reducing the motility of tumor cells, thus affecting the rate of invasion, (ii) preventing the formation of a favorable tumor microenvironment due to the possible effect on NETs, (iii) decreasing the level of SINE/LINE elements and c-Myc fragments, thus reducing the level of transformation and malignization, and (iv) inducing the apoptosis of tumor cells in metastatic foci. The contribution from all of these events at different levels of tumor progression (molecular, cellular and tissue) ultimately leads to antimetastatic activity of DNases.

## 4. Materials and Methods

### 4.1. Materials

Pulmozyme^®^ (Dornase alfa, 1000 U/mg protein, 1 mg/mL, Genotech Inc., San Francisco, CA, USA) in stock manufacture solution (8.772 mg/mL sodium chloride, 0.152 mg/mL calcium chloride dehydrate) and bovine pancreatic DNase I (Type IV, lyophilized powder, ≥2000 Kunitz units/mg, Sigma-Aldrich, Darmstadt, Germany) were used. 

### 4.2. Cell Cultures and Tumor Strains

Mouse melanoma B16 cell line was purchased from N.N. Blokhin Cancer Research Center (Moscow, Russia). B16 cells were grown in DMEM (Dulbecco’s Modified Eagle Medium, Thermo Fisher Scientific, Waltham, MA, USA) supplemented with 10% fetal bovine serum (FBS, MP Biomedicals, Irvine, CA, USA) and 1% antibiotic-antimycotic solution (10 mg/mL streptomycin, 10,000 U/mL penicillin, and 25 μg/mL amphotericin (MP Biomedicals, Irvine, CA, USA) at 37°C in a humidified atmosphere with 5% CO_2_. 

### 4.3. Mice

Male, 10–14-week-old C57Bl/6 (hereinafter, C57Bl) mice were obtained from the vivarium of ICBFM SB RAS (Novosibirsk, Russia). Mice were housed in plastic cages (ten animals per cage) under normal daylight conditions. Water and food were provided ad libitum. All animal procedures were carried out in strict accordance with the approved protocol and recommendations for proper use and care of laboratory animals (ECC Directive 2010/63/EU). The experimental protocols were approved by the Committee on the Ethics of Animal Experiments with the Institute of Cytology and Genetics SB RAS (ethical approval number 49 from 23 May 2019), and all efforts were made to minimize suffering.

At the start of the experiments, animal weight (mean ± SD) was 20.2 ± 1.5 g. Experimental groups contained 20 animals per group.

### 4.4. The Study of Deoxyribonuclease Activity of Pulmozyme^®^ and DNase I In Vitro

Pulmozyme^®^ and DNase I were dissolved in MilliQ water for *in vitro* experiments. DNase I reaction mixture (10 μL), containing 0.5 μg of plasmid pMDR670, DNase I (0.001–0.1 U), 10 mM Tris-HCl, pH 7.5, 2.5 mM MgCl_2_ and 0.1 mM CaCl_2_, was incubated at 37°C for 15 min. In the case of Pulmozyme^®^, reaction mixture (10 μL), containing 0.5 μg of plasmid pMDR670, Pulmozyme^®^ (0.001–0.1 U) and 2.5 mM MgCl_2_, no buffer, was incubated at the same conditions. After incubation, EDTA was added to final concentration of 2.5 mM, mixtures were kept at 65°C for 10 min, followed by extraction with phenol (pH 8.0) and chloroform. The cleavage products were analyzed by electrophoresis in 1% agarose gel followed by gel staining with ethidium bromide. The cleavage extent was calculated by equation [53]:

cleavage extent = ((p_relaxed_ + p_linearized_)/(p_relaxed_ + p_linearized_ + p_supercoiled_)) × 100%

where p_relaxed_—plasmid in relaxed form; p_linearized_—plasmid in linearized form and p_supercoiled_—supercoiled plasmid.

### 4.5. Influence of Pulmozyme^®^ and DNase I on cfDNA In Vitro 

The B16 cells (10 × 10^5^ cell/well, 24-well plate) were incubated in the presence of DNase I or Pulmozyme^®^ at the concentration 0.5 × 10^3^ U/mL in an FBS-free DMEM (500 μL) supplemented with 1% antibiotic-antimycotic solution for 24 h under standard conditions [54]. After incubation, the culture medium was collected from the monolayer culture of B16 cells; the cells were detached with TrypLE no phenol red (Thermo Fisher Scientific, Waltham, MA, USA), washed with PBS, and pelleted by centrifugation at 300 g rpm for 5 min, followed by washing with PBS and re-precipitation.

To remove cfDNA bound to the surface of cultured cells, 5 μL of 0.25% trypsin solution with EDTA was added to a cell suspension with a volume of 50 μL for 4 min. Then, a 5 μL of FBS was added to inhibit trypsin, and the mixture was centrifuged for 20 min at 350 g and 4°C. The supernatant was transferred to a new tube and centrifuged at 500 g for 20 min at 4°C to remove cells.

cfDNA from the cell surface and culture medium was isolated from the 0.1 mL of blood serum by extraction with phenol and chloroform, followed by concentration using ethanol precipitation. The concentration of cfDNA was measured by NanoDrop™ spectrophotometer (Thermo Fisher Scientific, Waltham, MA, USA) or Qubit fluorometer (Invitrogen, Thermo Fisher Scientific, Waltham, MA, USA) using a Quant-iT dsDNA HS Assay Kit (Invitrogen, Thermo Fisher Scientific, Waltham, MA, USA) according to the manufacturer’s recommendations.

### 4.6. Cell Viability Assay

The B16 cells (4 × 10^3^ cell/well, 96-well plate) were incubated in the presence of DNase I in the concentration range (1–0.75) × 10^3^ U/mL) or Pulmozyme^®^ (0.1–0.75 × 10^3^ U/mL) in an FBS-free DMEM (100 μL) supplemented with 1% antibiotic-antimycotic solution for 24 h under standard conditions [54]. Then, 3-(4, 5-dimethylthiazol-2-yl)-2, 5-diphenyltetrazolium bromide (MTT) (Sigma-Aldrich, Darmstadt, Germany) was added to each well at 0.5 mg/mL and cells were incubated for an additional 2 h. After that, the medium, containing MTT, was discarded from the wells and 100 µL of DMSO was added to each well to dissolve the formazan crystals, followed by measuring the optical density (OD) value at wavelengths ∆ (λ570–λ630) nm, on a Multiscan RC plate reader (Thermo LabSystems, Helsinki, Finland). Data are presented as percentage of living cells relative to control: (N_exp_/N_c_) × 100%. IC_20_ for Pulmozyme^®^ and DNase I were calculated as enzyme concentration at which 20% of cells died.

### 4.7. Apoptosis Assay

B16 cells were grown in 24-well plates in 500 µL DMEM supplemented with 10% FBS in the absence of antibiotics at a density of 1 × 10^5^ cells/well under standard conditions. On the day of experiment, the culture medium was replaced by fresh FBS-free DMEM supplemented with 1% antibiotic-antimycotic solution and DNase I (0.5 × 10^3^ U/mL) or Pulmozyme^®^ (0.5 × 10^3^ U/mL). Apoptosis analysis was performed using an Annexin V-FITC Apoptosis Staining/Detection Kit (Abcam, Cambridge, UK) according to the manufacturer’s protocols. Briefly, the cells were detached from the plate with Cell Dissociation Solution (Sigma-Aldrich, Darmstadt, Germany), washed with PBS and resuspended in 200 µL of 1× binding buffer supplemented with 2 µL of Annexin V-FITC and 2 µL of propidium iodide (for apoptosis analysis) or 2 µL of propidium iodide alone (for cell cycle analysis). The cells were incubated for 5 min at room temperature in the dark and analyzed on a NovoCyte Flow Cytometer (ACEA Biosciences, Santa Clara, CA, USA).

### 4.8. Scratch Assay (Wound-Healing)

B16 cells were grown in 6-well plates in 3 mL of DMEM supplemented with 10% FBS and 1% antibiotic-antimycotic solution per well until confluence was reached (1.0 × 10^6^ cell/well) under standard conditions. On the day of experiment, the culture medium was replaced by fresh FBS-free DMEM supplemented with 1% antibiotic-antimycotic solution and DNase I (0.1–1.5 × 10^3^ U/mL) or Pulmozyme^®^ (0.1–0.5 × 10^3^ U/mL). A wound was made by scratching the cells with a 200 μL pipette tip followed by cell incubation for 48 h under standard conditions. The scratched monolayer was photographed at 0, 2, 8, 24, 36 and 48 h after scratching using a Zeiss Primovert microscope (Zeiss, Oberkochen, Germany). Cell migration was assessed by measuring gap sizes at multiple fields using ImageJ 1.50f [55]. The migration length was estimated as the difference between scratch length filled with cells after 48 h and initial scratch length. The degree of scratch overgrowth was assessed by calculating using the equation:

υ = (1 − Χ) × 100%

where Χ is the ratio of the cell-free area of the scratch to the initial area of the scratch [14].

### 4.9. Tumor Implantation and Design of Animal Experiments

To generate a metastatic model of a melanoma B16 tumor, 1 × 10^5^ cells suspended in saline buffer (0.2 mL) were injected into the lateral tail vein of C57Bl mice. Pulmozyme^®^ and DNase I were dissolved in saline buffer for *in vivo* experiments.

#### 4.9.1. Intramuscular Experiment

On day 4 after tumor implantation, mice were assigned to 5 groups (*n* = 20): (1)—control, received saline buffer (0.1 mL); (2–4)—received Pulmozyme^®^ at the doses of 5, 25 and 50 U/mouse (0.1 mL), respectively; (5)—received DNase I at the dose 50 U/mouse (0.1 mL). Saline buffer, Pulmozyme^®^ or DNase I were administered intramuscularly daily except for weekends (5 + 2, total number of injections = 15).

#### 4.9.2. Intranasal Experiment

On day 5 after tumor implantation, mice were assigned to 3 groups (*n* = 20): (1)—control, received saline buffer (0.1 mL); (2)—received Pulmozyme^®^ at the dose 100 U/mice (0.1 mL); (3)—received DNase I at the dose 100 U/mice (0.1 mL). The intranasal administration was performed under isofluorane anesthesia. Saline buffer, Pulmozyme^®^ or DNase I were administered on days 5, 8, 12, 15, 19 and 22 (total number of administrations = 6). For one-day dynamic measurements on day 15 after tumor implantation, blood samples (0.1 mL) were collected from the retro-orbital sinus of 3 mice of each group at 0, 2, 4, 6 and 24 h time points after the administration of saline, Pulmozyme^®^ or DNase I for one-day dynamic measurement.

In both experiments on day 22, blood samples (up to 1 mL) were collected from the retro-orbital sinus 1 h after the last administration of saline, Pulmozyme^®^ and DNase I. Then, mice were sacrificed, and the organs occupied by metastases (lungs, liver) were isolated and fixed in 4% neutral-buffered formaldehyde (BioVitrum, St. Petersburg, Russia) for subsequent histological analysis. Surface metastases were counted using a binocular microscope. Internal metastases were analyzed using histology.

Blood samples of 5 healthy mice (0.2 mL) were collected from the retro-orbital sinus five times with an interval of 7 days. For one-day dynamic measurements, blood serum (0.1 mL) was taken from 3 healthy mice at 0, 2, 4, 6 and 24 h after the first blood sampling.

### 4.10. Histology and Immunohistochemistry

For histological evaluation of internal metastases, fixed lungs and livers were dehydrated in ascending ethanols and xylols, and embedded in HISTOMIX paraffin (BioVitrum, St. Petersburg, Russia). Paraffin sections (5 μm) were sliced on a Microm HM 355S microtome (Thermo Fisher Scientific, Waltham, MA, USA) and stained with hematoxylin and eosin. Images were obtained using an Axiostar Plus microscope equipped with an AxioCam MRc5 digital camera (Zeiss, Oberkochen, Germany). The percentages of the areas of internal metastases were determined relative to the total area of sections using Adobe Photoshop software at a magnification of ×100. The inhibition of internal metastasis development was assessed by morphometry using the metastasis inhibition index (MII), calculated as MII = [(mean metastasis area_control_ − mean metastasis area_experiment_)/mean metastasis area_control_] × 100%. The MII of the control group was taken as 0%, and the MII, corresponding to 100%, reflected the absence of metastases. Each studied group included 20 mice, and 10 to 15 random fields were studied in each specimen, forming 200–300 fields in total for each group of mice.

For DNA visualization, the lung sections (3 μm) were deparaffinized and rehydrated. The samples were incubated with the SYTO^TM^ 13 Green Fluorescent Nucleic Acid Stain (S7575, Invitrogen, Thermo Fisher Scientific, Waltham, MA, USA) according to the manufacturer’s protocol and embedded in Fluoromount-G^TM^ Mounting Medium (Invitrogen, Thermo Fisher Scientific, Waltham, MA, USA). For immunohistochemical study, the lung sections with metastases (3–5 μm) were deparaffnized and rehydrated. Antigen retrieval was carried out after exposure in a microwave oven at 700W. The samples were incubated with the Anti-Caspase-3 (ab2302, Abcam, Cambridge, UK), Anti-Caspase-7 (ab255818, Abcam, Cambridge, UK), Anti-Neutrophil (ab2557, Abcam, Cambridge, UK) or Anti-Neutrophil Elastase (ab68672) (Abcam, Cambridge, UK)-specific primary antibodies according to the manufacturer’s protocol. Then, the sections were incubated with secondary Alexa Fluor^®^ 488-conjugated antibodies (Invitrogen, Thermo Fisher Scientific, Waltham, MA, USA). and embedded in antifade mounting medium with DAPI VECTASHIELD^®^ (H-1200, Vector Laboratories, Inc., Burlingame, CA, USA).

SYTO^TM^ staining was assessed by confocal fluorescent microscopy on LSM710 (Zeiss, Oberkochen, Germany), using a plan-apochromat 63×/1.40 Oil DIC M27 objective. Analysis of SYTO^TM^ staining was performed using ZEN software (Zeiss, Oberkochen, Germany). Confocal analysis was performed in the green channel. Fluorescence in the green channel corresponded to the fluorescence of SYTO^TM^. Immunohistochemical images were examined and scanned using an Axiostar Plus microscope equipped with an HBO 50W/AC fluorescent lamp (Osram, Munich, Germany) and AxioCam MRc5 digital camera (Zeiss, Oberkochen, Germany) at a magnification of ×400. Images were processed using ZEN SP2 (Zeiss, Oberkochen, Germany) and ImageJ software. The intensity of green fluorescence, corresponding to the expression of caspase-3, caspase-7, neutrophils and neutrophil elastase, was calculated as corrected total fluorescence (CTF) according to the formula: CTF = integrated fluorescence density of metastasis − (metastasis area × mean fluorescence of background), and visualized as a heatmap using Morpheus tool.

### 4.11. Blood Serum Preparation

Blood serum was prepared from the whole blood by clot formation at 37 °C for 30 min and at 4 °C overnight, followed by clot discard, and centrifugation (4000 rpm, 4 °C, 20 min) to remove cell debris. Serum samples were stored at −70°C until use.

### 4.12. Measurement of DNase Activity in Blood Serum

The total DNase activity of blood serum was evaluated in a cleavage reaction of plasmid pSVK [56]. The reaction mixture (30 μL) containing 1 μL of serum and 0.5 μg of plasmid in MilliQ H_2_O was incubated at 30 °C for 5–30 min. After incubation, EDTA was added to a final concentration of 2.5 mM, and mixtures were kept at 65 °C for 10 min, followed by extraction with phenol (pH 8.0) and chloroform. The cleavage products were analyzed by electrophoresis in a 1% agarose gel followed by gel staining with ethidium bromide. The effective cleavage rate constants (k_eff_) were obtained using the equation: P_t_ = P_∞_ × (1 − exp^−keff t^), where P_t_ and P_∞_ correspond to the fraction of the substrate digested at time t and at the end point, respectively [53].

### 4.13. Isolation of cfDNA from Blood Serum

cfDNA was isolated from the 0.1 mL of blood serum by extraction with phenol and chloroform, followed by concentration using ethanol precipitation. The concentration of cfDNA was measured by NanoDrop™ spectrophotometer (Thermo Scientific, Waltham, MA, USA) or Qubit fluorometer (Invitrogen, Thermo Fisher Scientific, Waltham, MA, USA)using a Quant-iT dsDNA HS Assay Kit (Invitrogen, Thermo Fisher Scientific, Waltham, MA, USA) according to the manufacturer’s recommendations.

### 4.14. Real-Time RT-PCR

The levels of B1_mus2, L1_mus1 and c-Myc fragments in cfDNA from blood serum were measured by SYBR-Green-based real-time qPCR. The PCR mixture (20 μL) contained 5 μL of cfDNA solution (0.1–0.5 ng per reaction), 10 μL of SYBR-Green-containing BioMasterCor HS-qPCR (BiolabMix, Novosibirsk, Russia) and 0.6 μmol of each primer. The primer sequences are listed in Table S13 (Supplementary materials). The PCR was performed using CFX96 Touch Real-Time PCR Detection System (Bio-Rad Laboratories Inc., Hercules, CA, USA). The cycling conditions were as follows: 95 °C, 6 min; 95 °C, 15 s; 60 °C, 20 s; 70 °C, 60 s; 40 cycles; followed by melt curve analysis [20]. The PCR results were normalized to the level of β-actin and expressed as arbitrary unit (a.u.). PCR specificity was controlled using melting curve analysis. Calculation of fragment level normalized to β-actin was carried out according to the comparative threshold cycle (∆∆*CT*) method.

### 4.15. Statistics

Correlation analysis was performed using multiple regression order correlation coefficient (R^2^), which is the correlation coefficient between the observed values of the outcome variable and the fitted (i.e., predicted) values, and Spearman’s rank (r), which is a statistical measure of the strength of the relationship between paired data. The relationship between studied parameters (variables) was estimated taking the values of Spearman’s coefficient or regression analysis coefficient equal to 0.01 ≤ r or R^2^ < 0.3 as being indicative of a weak correlation, 0.3 ≤ r or R^2^ < 0.7 as a moderate correlation, and 0.70 ≤ r or R^2^ ≤ 0.99 as a strong correlation. Positive and negative values indicate positive and negative correlations. 

All experiments were reproduced in triplicate. Data of MTT and the scratch assay were statistically processed using Student’s t-test (two tailed, unpaired). MII, metastasis number, DNase activity, cfDNA concentration and PCR data were statistically processed using one-way ANOVA. Post hoc testing was completed using a post hoc Tukey test; *p* < 0.05 was considered to be statistically significant. The statistical package STATISTICA version 10.0 was used for analysis.

## 5. Conclusions

In conclusion, it was shown that human recombinant DNase I in the form of Pulmozyme^®^ is a powerful tool for the inhibition of lung metastases, and its antimetastatic activity correlated with the restoration of DNase activity, a decrease in cfDNA concentrations and the specific degradation of the Myc oncogene and SINE and LINE elements. Pulmozyme^®^ substantially outperforms the effects of bovine pancreatic DNase I when applied in an intranasal form. This work represents the first step towards a clinical application of Pulmozyme^®^ as antimetastatic therapeutic. Undoubtedly, a wide range of *in vitro* and *in vivo* studies is required to confirm the effectiveness of Pulmozyme^®^ in the treatment of human tumors metastasized to lungs. In addition, further research is needed to confirm cfDNA as the main target of Pulmozyme^®^ and to elucidate their role (cfDNA) in tumor progression.

## Supplementary Materials

The following are available online at https://www.mdpi.com/article/10.3390/ijms222112074/s1.
