# Supplementary material for: Human Recombinant DNase I (Pulmozyme®) Inhibits Lung Metastases in Murine Metastatic B16 Melanoma Model That Correlates with Restoration of the DNase Activity and the Decrease SINE/LINE and c-Myc Fragments in Blood Cell-Free DNA
Source: Int J Mol Sci. 2021 Nov 8;22(21):12074. doi: 10.3390/ijms222112074 (PMC8585023; doi:10.3390/ijms222112074)
Supplement: Supplementary file 1 [file ijms-22-12074-s001.zip › ijms-1417378-supplementary.pdf]

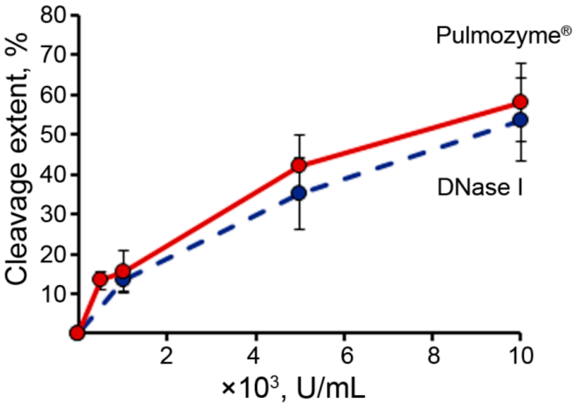

Figure S1. Concentration dependence of plasmid pMDR670 cleavage by Pulmozyme® and DNase I. Pulmozyme® or DNase I (0.001 – 0.1 U) were incubated with plasmid pMDR670 at 37°C for 15 min. Cleavage products were analysed by electrophoresis in 1% agarose gel and stained by ethidium bromide. Cleavage extent was calculated as described in Materials and Methods.

Table S1. The effect of Pulmozyme® and DNase I on the cfDNA concentration on the cell surface and in culture medium of B16 melanoma cells.

cfDNA	Control	DNase I	Pulmozyme®
**On the surface**	62 ± 11	4 ± 1	3 ± 1
**In the culture medium**	2226 ±942	142 ± 65	189 ± 95

The B16 cells were incubated in the presence of DNase I or Pulmozyme® at the concentration 0.5 × 10^3 U/mL in a FBS-free DMEM supplemented with 1% antibiotic-antimycotic solution for 24 h under standard conditions. cfDNAs bound to the surface of cultured cells and cfDNA from culture medium were collected. Data are presented as mean. Data were statistically analysed using one-way ANOVA with a post hoc Tukey test.

Table S2. The concentration of cfDNA in blood serum of mice with B16 treated with Pulmozyme® and DNase I.

Type of administration	cfDNA, ng/mL			
	**H**	**B16**	**Pulmozyme®**	**DNase I**
**i.m.**			**50 U/mouse**	
	630±94	1190±87	1463±303	676±71
**i.n.**			**100 U/mouse**	
	567±94	1180±343	765±122	1285±152

See Figure 2A (for i.m.) and Figure 3A (for i.n.) for experimental setup. B16 cells (5 × 10^5 cells/mL, 0.2 mL) were implanted intravenously (i.v.) into C57Bl/6J mice (male). I.m. experiment: starting from the day 4 animals received i.m. saline buffer, Pulmozyme® (5, 25 or 50 U/mouse) or DNase I (50 U/mouse) daily except for weekend. I.n. experiment: starting from the day 5 animals received i.n. saline buffer, Pulmozyme® (100 U/mouse) or DNase I (100 U/mouse) twice a week. On the day 15, 1 h after the administration, blood sampling was performed, and cfDNA was isolated. Data are presented as median. Data were statistically analysed using one-way ANOVA with a post hoc Tukey test. H – healthy animals; B16, DNase I and Pulmozyme® – mice with B16 received saline buffer,– DNase I or Pulmozyme®.

Table S3. DNase activity in blood serum of mice with B16 treated with Pulmozyme® and DNase I.

Type of administration	DNase activity (k_{eff}, ×10^{-3} s^{-1})			
	**H**	**B16**	**Pulmozyme®**	**DNase I**
**i.m.**			**50 U/mouse**	
	0.62±0.02	0.22±0.008	0.44±0.02	0.64±0.04
**i.n.**			**100 U/mouse**	
	0.62±0.02	0.26±0.03	5.95±0.24	1.20±0.22

See Figure 2A (for i.m.) and Figure 3A (for i.n.) for experimental setup. On the day 15, 1 h after the administration, blood sampling was performed, and DNase activity in blood serum was measured. Data are presented as median. Data were statistically analysed using one-way ANOVA with a post hoc Tukey test. H – healthy animals; B16, DNase I and Pulmozyme® – mice with B16 received saline buffer,– DNase I or Pulmozyme®.

Table S4. The level of B1_mus2 in blood serum of mice with B16 treated with saline buffer, Pulmozyme® and DNase I.

Type of administration	B1_mus2/b-actin, a.u.			
	**H**	**B16**	**Pulmozyme®**	**DNase I**
**i.m.**			**50 U/mouse**	
	0.13±0.01	70±14	3±1.9	3.3±1
**i.n.**			**100 U/mouse**	
	0.13±0.01	1154±753	33±30	303±97

See Figure 2A (for i.m.) and Figure 3A (for i.n.) for experimental setup. On the day 15, 1 h after the administration, blood sampling was performed, cfDNA was isolated, and the level of B1_mus2 fragments was measured by RT-qPCR. The PCR meanings were normalized to the level of β-actin and expressed as arbitrary unit (a.u.). Data are presented as median. Data were statistically analysed using one-way ANOVA with a post hoc Tukey test. H – healthy animals; B16, DNase I and Pulmozyme® – mice with B16 received saline buffer,– DNase I or Pulmozyme®.

Table S5. The level of L1_mus1 in blood serum of mice with B16 treated with saline buffer, Pulmozyme® and DNase I.

Type of administration	L1_mus1/b-actin, a.u.			
	**H**	**B16**	**Pulmozyme®**	**DNase I**
**i.m.**			**50 U/mouse**	
	1.12±0.15	54.3±11	5±3.6	4±1.9
**i.n.**			**100 U/mouse**	
	1.12±0.15	598±152	33±30	108±51

See Figure 2A (for i.m.) and Figure 3A (for i.n.) for experimental setup. On the day 15, 1 h after the administration, blood sampling was performed, cfDNA was isolated, and the level of L1_mus2 fragments was measured by RT-qPCR. The PCR meanings were normalized to the level of β-actin and expressed as arbitrary unit (a.u.). Data are presented as median. Data were statistically analysed using one-way ANOVA with a post hoc Tukey test. H – healthy animals; B16, DNase I and Pulmozyme® – mice with B16 received saline buffer,– DNase I or Pulmozyme®.

Table S6. The level of c-Myc in blood serum of mice with B16 treated with saline buffer, Pulmozyme® and DNase I.

Type of administration	c-Myc/b-actin, a.u.			
	**H**	**B16**	**Pulmozyme®**	**DNase I**
**i.m.**			**50 U/mouse**	
	0.36±0.07	69±7.4	12.5±2.7	6.6±2.5
**i.n.**			**100 U/mouse**	
	0.36±0.07	738±231	2±2	114.2±61

See Figure 2A (for i.m.) and Figure 3A (for i.n.) for experimental setup. On the day 15, 1 h after the administration, blood sampling was performed, cfDNA was isolated, and the level of c-Myc fragments was measured by RT-qPCR. The PCR meanings were normalized to the level of β-actin and expressed as arbitrary unit (a.u.). Data are presented as median. Data were statistically analysed using one-way ANOVA with a post hoc Tukey test. H – healthy animals; B16, DNase I and Pulmozyme® – mice with B16 received saline buffer,– DNase I or Pulmozyme®.

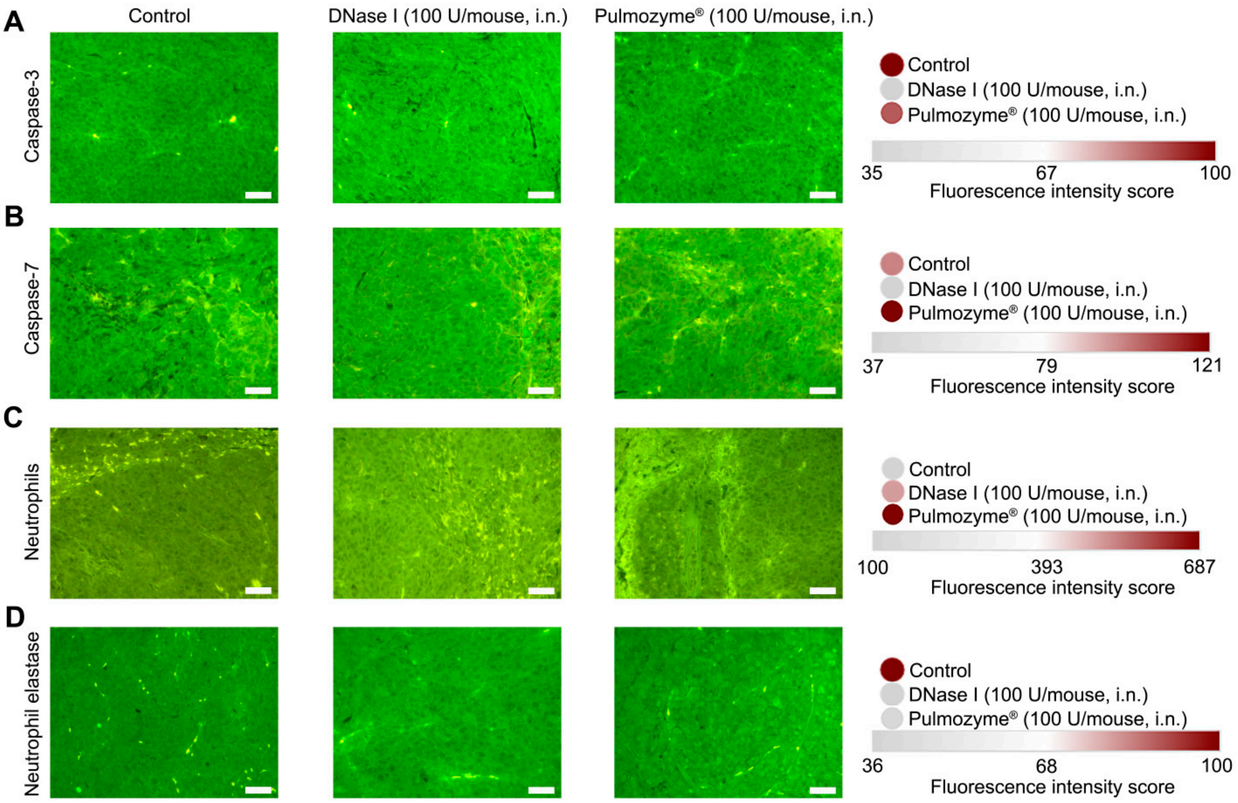

Figure S2. Pulmozyme® enhances the expression of apoptosis- and neutrophil-associated markers in B16 melanoma metastatic foci. Representative images of the fluorescence-based immunohistochemical staining of lung metastases with anti-Caspase-3 (**A**), anti-Caspase-7 (**B**), anti-Neutrophil (**C**), and anti-Neutrophil Elastase (**D**) primary antibodies with subsequent incubation with secondary Alexa Fluor® 488-conjugated antibodies. Scale bar corresponds to 50 μm. The intensity of green fluorescence, corresponding to the expression of Caspase-3, Caspase-7, Neutrophils, and Neutrophil Elastase, was calculated for each image using ImageJ software, normalized to the brightness of the control samples and visualized as a heatmap using Morpheus tool.

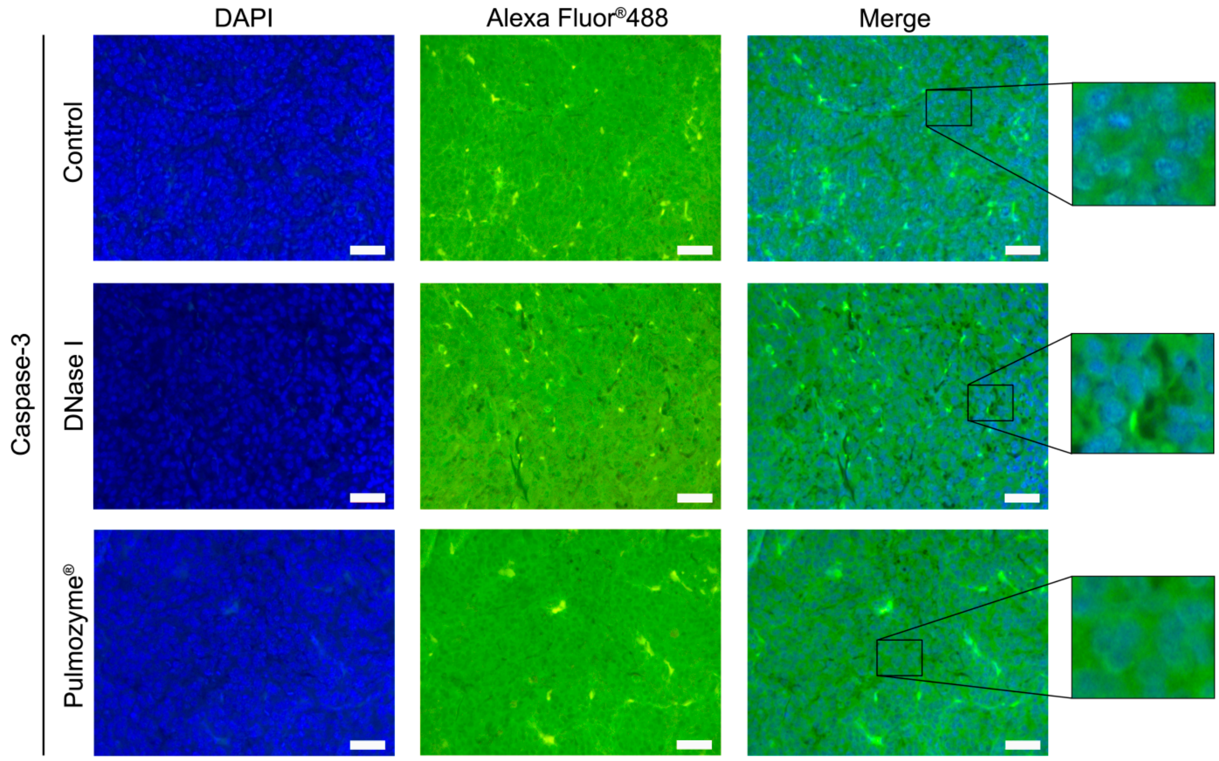

Figure S3. Representative images of the fluorescence-based immunohistochemical staining of lung metastases with anti-Caspase-3 primary antibodies with subsequent incubation with secondary Alexa Fluor® 488-conjugated antibodies, embedded in antifade mounting medium with DAPI Vectashield®. Scale bar corresponds to 50 μm.

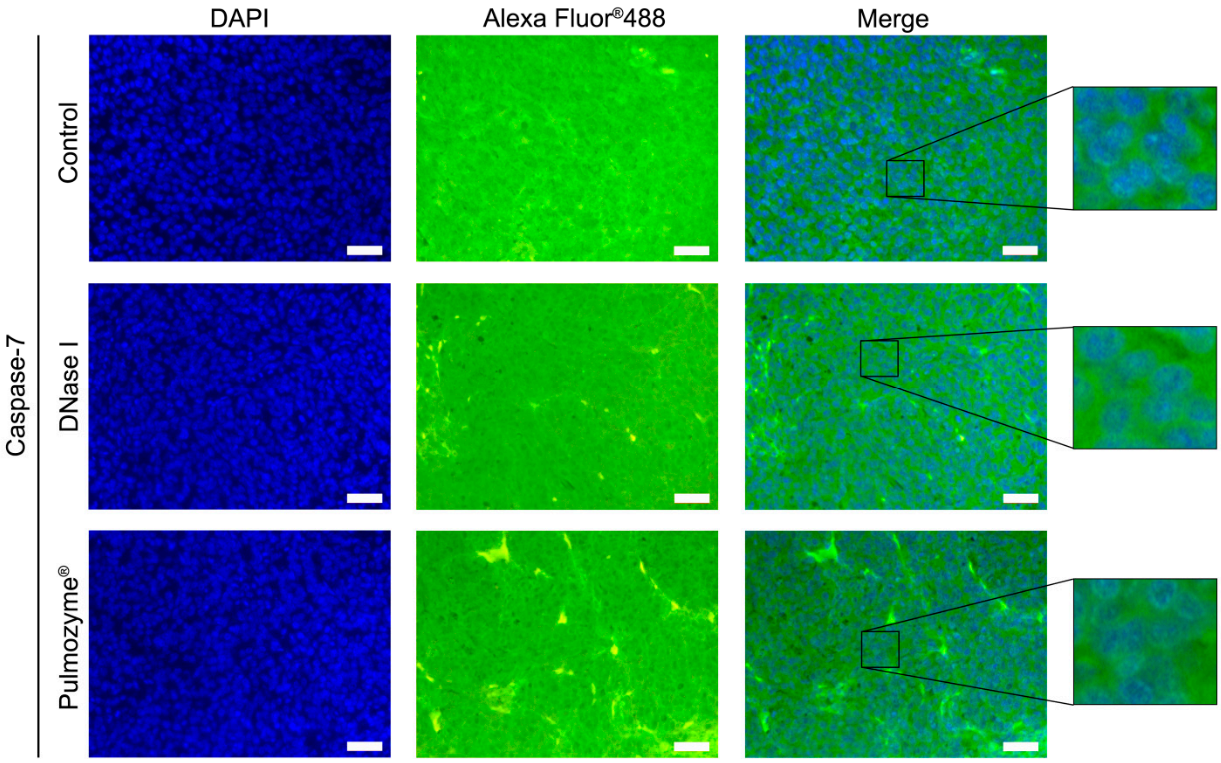

Figure S4. Representative images of the fluorescence-based immunohistochemical staining of lung metastases with anti-Caspase-7 primary antibodies with subsequent incubation with secondary Alexa Fluor® 488-conjugated antibodies, embedded in antifade mounting medium with DAPI Vectashield®. Scale bar corresponds to 50 μm.

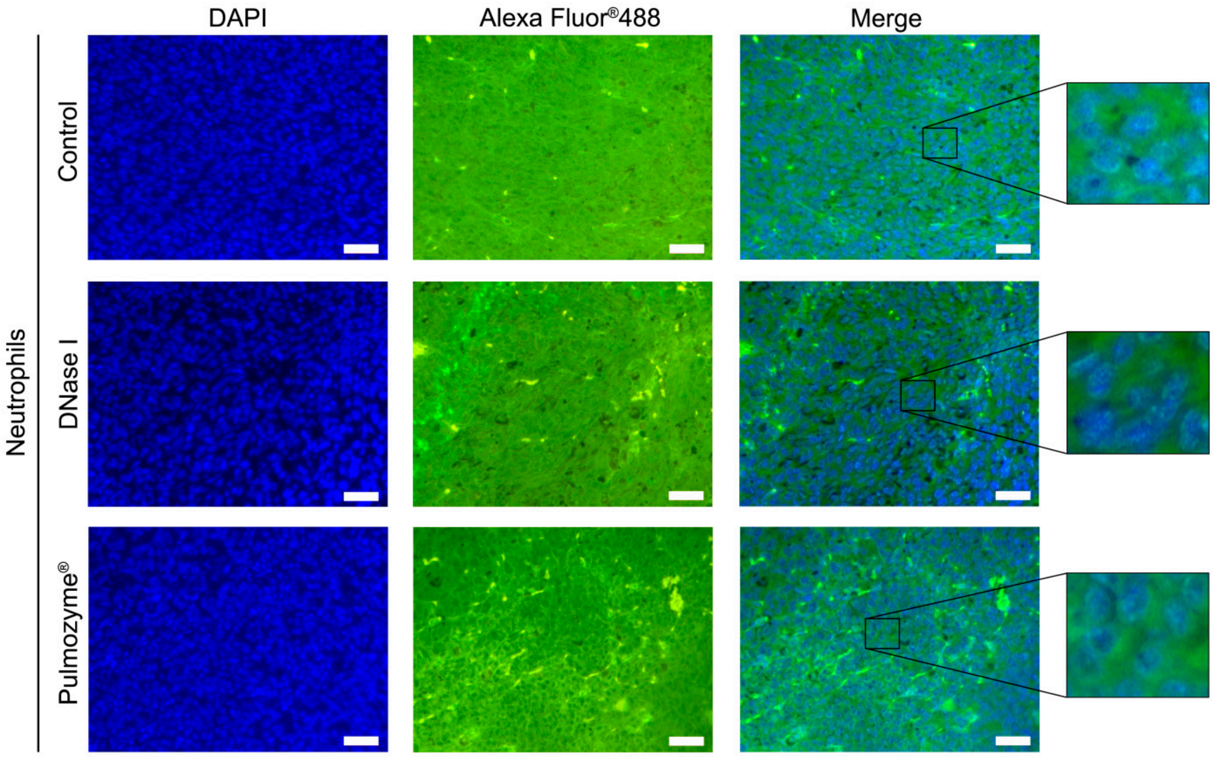

Figure S5. Representative images of the fluorescence-based immunohistochemical staining of lung metastases with anti-Neutrophils primary antibodies with subsequent incubation with secondary Alexa Fluor® 488-conjugated antibodies, embedded in antifade mounting medium with DAPI Vectashield®. Scale bar corresponds to 50 μm.

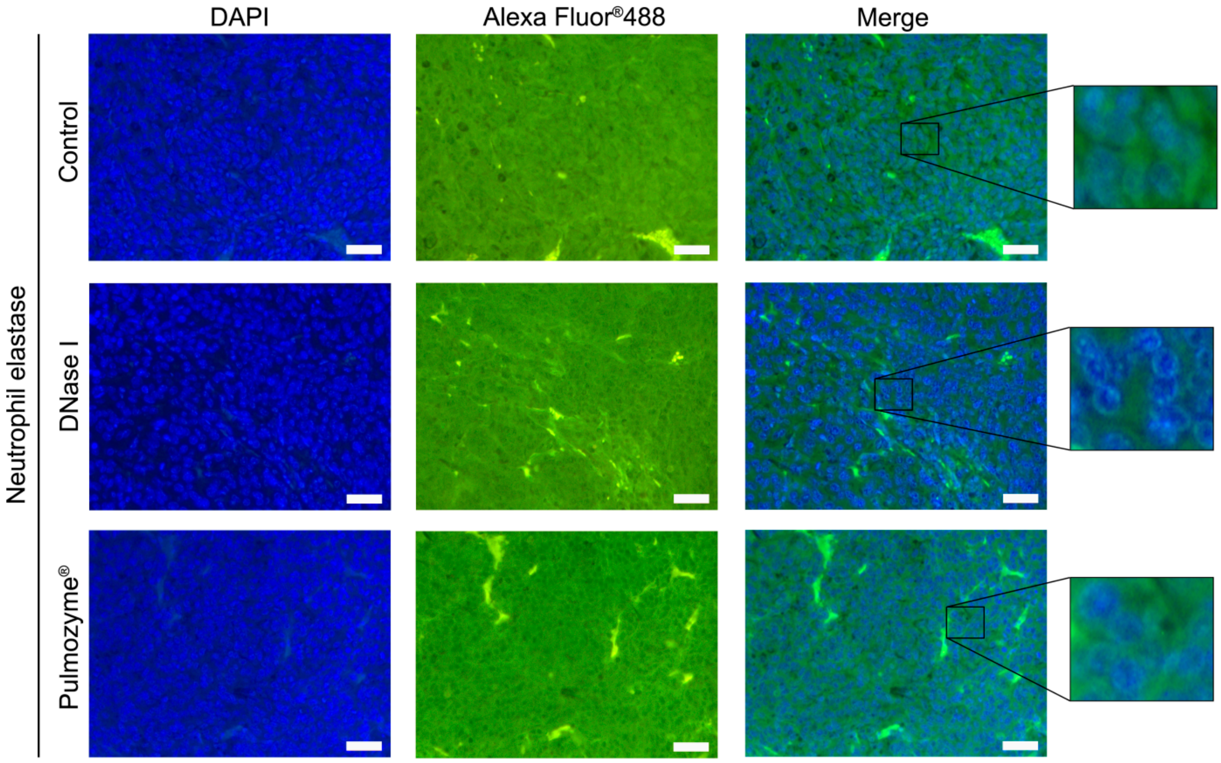

Figure S6. Representative images of the fluorescence-based immunohistochemical staining of lung metastases with anti-Neutrophil Elastase primary antibodies with subsequent incubation with secondary Alexa Fluor® 488-conjugated antibodies, embedded in antifade mounting medium with DAPI Vectashield®. Scale bar corresponds to 50 μm.

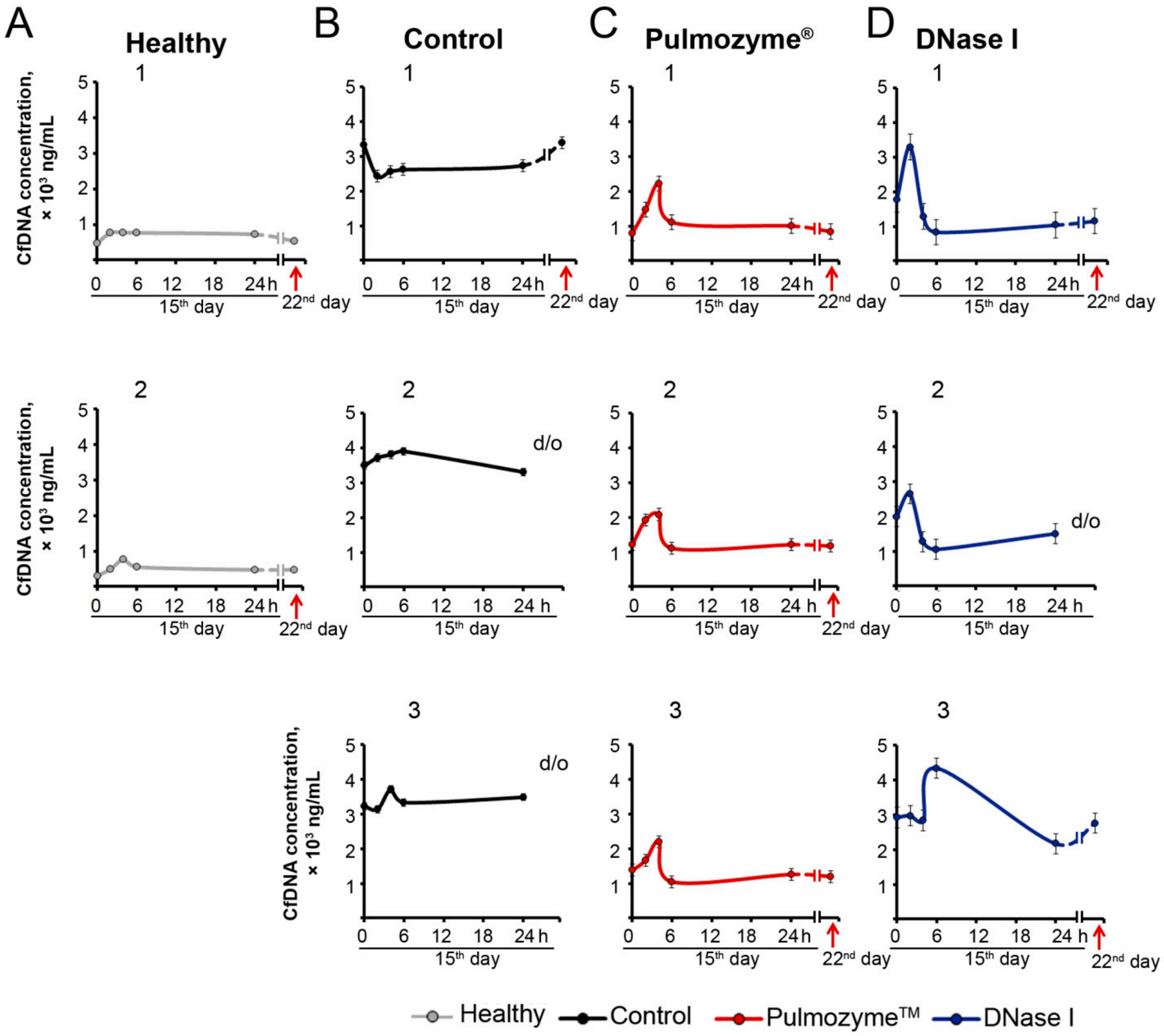

Figure S7. The one day dynamic of cfDNA concentration in blood serum of mice with metastatic B16 after the i.n. treatment with Pulmozyme® or DNase I. Treatment scheme is presented in Figure 3A. On the day 15 after B16 implantation after i.n. administration of saline buffer, Pulmozyme® or DNase I, blood sampling was performed at 0, 2, 4, 6 and 24 h. **A.** Healthy mice (n=2). **B, C and D.** Mice with B16 treated with saline buffer (n=3); Pulmozyme® (n=3) and DNase I (n=3), respectively. Data are presented as mean ± SE. Healthy group is marked by grey, control group – by black, DNase I group – by blue, Pulmozyme® group – by red. d/o – mice dropped out of the experiment.

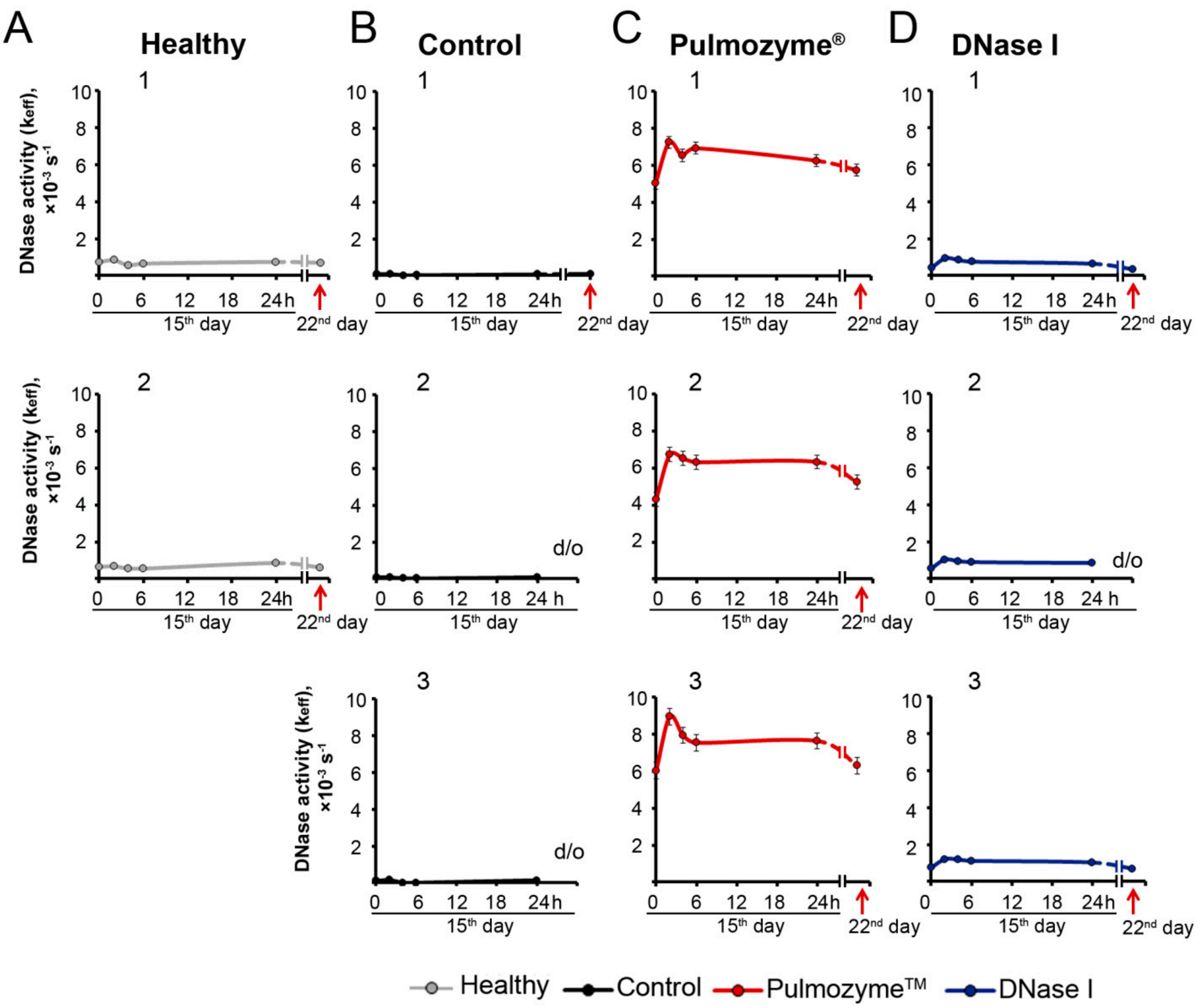

Figure S8. The one day dynamic of DNase activity in blood serum of mice with metastatic B16 after the i.n. treatment with Pulmozyme® or DNase I. Treatment scheme is presented in Figure 3A. On the day 15 after B16 implantation after i.n. administration of saline buffer (control), Pulmozyme® and DNase I, blood sampling was performed at 0, 2, 4, 6 and 24 h time points. **A.** Healthy mice (n=2). **B, C** and **D.** Mice with B16 treated with saline buffer (n=3), Pulmozyme® (n=3) and DNase I (n=3), respectively Data are presented as mean ± SE. Healthy group is marked by grey, control group – by black, DNase I group – by blue, Pulmozyme® group – by red. d/o – mice dropped out of the experiment.

Table S7. The multiple regression correlation coefficients for different variables for mice injected i.m. by DNase I or Pulmozyme®.

	Correlations: i.m. injections					
**Variable**	**[cfDNA]**	**DNase activity**	**C-Myc**	**B1_mus2**	**L1_mus1**	**Lung metastases**
**[cfDNA]**		-0.42	0.54	0.60	0.59	0.59
**DNase activity**	-0.42		-0.42	-0.48	-0.42	-0.59
**C-Myc**	0.54	-0.42		0.91	0.93	0.59
**B1_mus2**	0.60	-0.48	0.91		0.98	0.59
**L1_mus1**	0.60	-0.42	0.93	0.98		0.58
**Lung metastases**	0.60	-0.59	0.59	0.60	0.58	

[cfDNA] – concentration of cfDNA; DNase activity - k_{eff}; c-Myc, B1_mus2 and L1_mus1 – level of specific fragments normalized to β–actin; lung metastases – number of lung metastases. The values marked by red shows strong correlations (0.7 – 0.99); marked by blue – medium correlations (0.3 – 0.7 not inclusive); marked by black – low correlations (0.01 – 0.3 not inclusive).

Table S8. The multiple regression summary for different variables for mice injected i.m. by DNase I or Pulmozyme®.

	Regression Summary for Dependent Variable: Lung metastases, i.m. injections $R = 0.74$; $R^2 = 0.55$; $p < 0.0005$		
**Variable**	**β**	**St.err for β**	**p-value**
**[cfDNA]**	0.30	0.15	0.0563
**DNase activity**	**-0.37**	**0.14**	**0.0161**
**B1_mus2**	-0.16	0.64	0.8041
**L1_mus1**	0.08	0.68	0.9021
**C-Myc**	0.35	0.31	0.2761

[cfDNA] – concentration of cfDNA; DNase activity - k_{eff}; C-Myc, B1_mus2 and L1_mus1 – level of specific fragments normalized to β–actin. The number of lung metastases was used as independent variable. β is the partial correlation coefficients. The values marked bold are significant at $p < 0.05$.

Table S9. The Spearman correlation coefficients for different variables for mice injected i.m. by DNase I or Pulmozyme®.

	Spearman Rank Order Correlations: i.m. injections					
**Variable**	**Lung metastases**	**B1_mus2**	**L1_mus1**	**C-Myc**	**[cfDNA]**	**DNase activity**
**Lung metastases**		**-0.42**	**0.54**	**0.60**	**0.59**	**0.59**
**B1_mus2**	**-0.42**		**-0.42**	**-0.48**	**-0.42**	**-0.59**
**L1_mus1**	**0.54**	**-0.42**		**0.91**	**0.93**	**0.59**
**C-Myc**	**0.60**	**-0.48**	**0.91**		**0.98**	**0.59**
**[cfDNA]**	**0.60**	**-0.42**	**0.93**	**0.98**		**0.58**
**DNase activity**	**0.59**	**-0.59**	**0.59**	**0.59**	**0.58**	

[cfDNA] – concentration of cfDNA; DNase activity - k_{eff}; C-Myc, B1_mus2 and L1_mus1 – level of specific fragments normalized to β–actin; lung metastases – number of lung metastases. The values marked by red shows strong correlations (0.7 – 0.99); marked by blue – medium correlations (0.3 – 0.7 not inclusive); marked by black – low correlations (0.01 – 0.3 not inclusive).

Table S10. The multiple regression correlation coefficients for different variables for mice administrated i.n. by DNase I or Pulmozyme®.

	Correlations: i.n. administration						
**Variable**	**Lung metastases**	**[cfDNA]**	**DNase activity**	**B1_mus2**	**L1_mus1**	**C-Myc**	**Liver metastases**
**Lung metastases**		0.63	-0.65	0.21	0.27	0.32	0.57
**[cfDNA]**	0.63		-0.32	0.07	0.11	0.25	0.22
**DNase activity**	-0.65	-0.32		-0.20	-0.25	-0.49	-0.39
**B1_mus2**	0.21	0.07	-0.20		0.97	0.05	-0.03
**L1_mus1**	0.27	0.11	-0.25	0.97		0.11	0.01
**C-Myc**	0.32	0.25	-0.49	0.05	0.11		0.18
**Liver metastases**	0.57	0.22	-0.39	-0.03	0.01	0.18	

[cfDNA] – concentration of cfDNA; DNase activity - k_{eff}; C-Myc, B1_mus2 and L1_mus1 – level of specific fragments normalized to β–actin; lung metastases – number of lung metastases; liver metastases – number of liver metastases. The values marked by red shows strong correlations (0.7 – 0.99); marked by blue – medium correlations (0.3 – 0.7 not inclusive); marked by black – low correlations (0.01 – 0.3 not inclusive).

Table S11. The multiple regression summary for different variables for mice administrated i.n. by DNase I or Pulmozyme®.

	Regression Summary for Dependent Variable: Lung metastases, i.n. administration $R= 0.94$; $R^2= 0.89$; $p < 0.00001$		
**Variable**	**β**	**St.err for β**	**p-value**
**[cfDNA]**	**0.36**	**0.14**	**0.0189**
**DNase activity**	-0.10	0.13	0.4862
**B1_mus2**	**-2.18**	**0.62**	**0.0022**
**L1_mus1**	**2.38**	**0.63**	**0.0011**
**C-Myc**	**-0.34**	**0.13**	**0.0182**
**Liver metastases**	**0.41**	**0.09**	**0.0002**

[cfDNA] – concentration of cfDNA; DNase activity - k_{eff}; C-Myc, B1_mus2 and L1_mus1 – level of specific fragments normalized to β–actin; liver metastases – number of liver metastases. The number of lung metastases was used as independent variable. β is the partial correlation coefficient. The values marked bold are significant at $p < 0.05$.

Table S12. The Spearman correlation coefficients for different variables for mice administrated i.n. by DNase I or Pulmozyme®.

	Spearman Rank Order Correlations: i.n. administration Bold Marked correlations are significant at $p < 0.05$						
**Variable**	**Lung metastases**	**Liver metastases**	**[cfDNA]**	**DNase activity**	**B1_mus2**	**L1_mus1**	**C-Myc**
**Lung metastases**		**0.51**	**0.54**	**-0.82**	**0.51**	**0.46**	**0.60**
**Liver Metastases**	**0.51**		0.26	**-0.40**	0.12	0.05	0.19
**[cfDNA]**	**0.54**	0.26		**-0.43**	0.26	**0.38**	**0.35**
**DNase activity**	**-0.82**	**-0.40**	**-0.43**		**-0.61**	**-0.51**	**-0.67**
**B1_mus2**	**0.51**	0.12	0.26	**-0.61**		**0.89**	**0.83**
**L1_mus1**	**0.46**	0.05	**0.38**	**-0.51**	**0.89**		**0.84**
**C-Myc**	**0.60**	0.19	**0.35**	**-0.67**	**0.83**	**0.84**	

[cfDNA] – concentration of cfDNA; DNase activity - k_{eff}; C-Myc, B1_mus2 and L1_mus1 – level of specific fragments normalized to β–actin; lung metastases – number of lung metastases; liver metastases – number of liver metastases. The values marked by red shows strong correlations (0.7 – 0.99); marked by blue – medium correlations (0.3 – 0.7 not inclusive); marked by black – low correlations (0.01 – 0.3 not inclusive). The values marked bold are significant at $p < 0.05$.

Table S13. Primer sequences used for real-time PCR.

Repeat type	Primer sequences 5′ – 3′
B1_mus2_F	GAGACAGGCGGATTTCTGAGT
B1_mus2_R	TGTAGCCCTGGCTGTCCT
L1_mus1_F	GCCAGGTATCTGTGCATCTT
L1_mus1_R	ACTCTAGCTCTCTCCTGAGTTT
Myc F	CGACTACGACTCCGTACAGC
Myc R	CCAGATATCCTCACTGGGCG
β-actin F	TCTTTGCAGCTCCTTCGTTG
β-actin R	AGTGAGGTACTAGCCACGAGA
